# Author Correction: Discovery of widespread transcription initiation at microsatellites predictable by sequence-based deep neural network

**DOI:** 10.1038/s41467-022-28758-y

**Published:** 2022-03-01

**Authors:** Mathys Grapotte, Manu Saraswat, Chloé Bessière, Christophe Menichelli, Jordan A. Ramilowski, Jessica Severin, Yoshihide Hayashizaki, Masayoshi Itoh, Michihira Tagami, Mitsuyoshi Murata, Miki Kojima-Ishiyama, Shohei Noma, Shuhei Noguchi, Takeya Kasukawa, Akira Hasegawa, Harukazu Suzuki, Hiromi Nishiyori-Sueki, Martin C. Frith, FANTOM consortium, Imad Abugessaisa, Stuart Aitken, Bronwen L. Aken, Intikhab Alam, Tanvir Alam, Rami Alasiri, Ahmad M. N. Alhendi, Hamid Alinejad-Rokny, Mariano J. Alvarez, Robin Andersson, Takahiro Arakawa, Marito Araki, Taly Arbel, John Archer, Alan L. Archibald, Erik Arner, Peter Arner, Kiyoshi Asai, Haitham Ashoor, Gaby Astrom, Magda Babina, J. Kenneth Baillie, Vladimir B. Bajic, Archana Bajpai, Sarah Baker, Richard M. Baldarelli, Adam Balic, Mukesh Bansal, Arsen O. Batagov, Serafim Batzoglou, Anthony G. Beckhouse, Antonio P. Beltrami, Carlo A. Beltrami, Nicolas Bertin, Sharmodeep Bhattacharya, Peter J. Bickel, Judith A. Blake, Mathieu Blanchette, Beatrice Bodega, Alessandro Bonetti, Hidemasa Bono, Jette Bornholdt, Michael Bttcher, Salim Bougouffa, Mette Boyd, Jeremie Breda, Frank Brombacher, James B. Brown, Carol J. Bult, A. Maxwell Burroughs, Dave W. Burt, Annika Busch, Giulia Caglio, Andrea Califano, Christopher J. Cameron, Carlo V. Cannistraci, Alessandra Carbone, Ailsa J. Carlisle, Piero Carninci, Kim W. Carter, Daniela Cesselli, Jen-Chien Chang, Julie C. Chen, Yun Chen, Marco Chierici, John Christodoulou, Yari Ciani, Emily L. Clark, Mehmet Coskun, Maria Dalby, Emiliano Dalla, Carsten O. Daub, Carrie A. Davis, Michiel J. L. de Hoon, Derek de Rie, Elena Denisenko, Bart Deplancke, Michael Detmar, Ruslan Deviatiiarov, Diego Di Bernardo, Alexander D. Diehl, Lothar C. Dieterich, Emmanuel Dimont, Sarah Djebali, Taeko Dohi, Jose Dostie, Finn Drablos, Albert S. B. Edge, Matthias Edinger, Anna Ehrlund, Karl Ekwall, Arne Elofsson, Mitsuhiro Endoh, Hideki Enomoto, Saaya Enomoto, Mohammad Faghihi, Michela Fagiolini, Mary C. Farach-Carson, Geoffrey J. Faulkner, Alexander Favorov, Ana Miguel Fernandes, Carmelo Ferrai, Alistair R. R. Forrest, Lesley M. Forrester, Mattias Forsberg, Alexandre Fort, Margherita Francescatto, Tom C. Freeman, Martin Frith, Shinji Fukuda, Manabu Funayama, Cesare Furlanello, Masaaki Furuno, Chikara Furusawa, Hui Gao, Iveta Gazova, Claudia Gebhard, Florian Geier, Teunis B. H. Geijtenbeek, Samik Ghosh, Yanal Ghosheh, Thomas R. Gingeras, Takashi Gojobori, Tatyana Goldberg, Daniel Goldowitz, Julian Gough, Dario Greco, Andreas J. Gruber, Sven Guhl, Roderic Guigo, Reto Guler, Oleg Gusev, Stefano Gustincich, Thomas J. Ha, Vanja Haberle, Paul Hale, Bjrn M. Hallstrom, Michiaki Hamada, Lusy Handoko, Mitsuko Hara, Matthias Harbers, Jennifer Harrow, Jayson Harshbarger, Takeshi Hase, Akira Hasegawa, Kosuke Hashimoto, Taku Hatano, Nobutaka Hattori, Ryuhei Hayashi, Yoshihide Hayashizaki, Meenhard Herlyn, Peter Heutink, Winston Hide, Kelly J. Hitchens, Shannon Ho Sui, Peter A. C. ’t Hoen, Chung Chau Hon, Fumi Hori, Masafumi Horie, Katsuhisa Horimoto, Paul Horton, Rui Hou, Edward Huang, Yi Huang, Richard Hugues, David Hume, Hans Ienasescu, Kei Iida, Tomokatsu Ikawa, Toshimichi Ikemura, Kazuho Ikeo, Norihiko Inoue, Yuri Ishizu, Yosuke Ito, Masayoshi Itoh, Anna V. Ivshina, Boris R. Jankovic, Piroon Jenjaroenpun, Rory Johnson, Mette Jorgensen, Hadi Jorjani, Anagha Joshi, Giuseppe Jurman, Bogumil Kaczkowski, Chieko Kai, Kaoru Kaida, Kazuhiro Kajiyama, Rajaram Kaliyaperumal, Eli Kaminuma, Takashi Kanaya, Hiroshi Kaneda, Philip Kapranov, Artem S. Kasianov, Takeya Kasukawa, Toshiaki Katayama, Sachi Kato, Shuji Kawaguchi, Jun Kawai, Hideya Kawaji, Hiroshi Kawamoto, Yuki I. Kawamura, Satoshi Kawasaki, Tsugumi Kawashima, Judith S. Kempfle, Tony J. Kenna, Juha Kere, Levon Khachigian, Hisanori Kiryu, Mami Kishima, Hiroyuki Kitajima, Toshio Kitamura, Hiroaki Kitano, Enio Klaric, Kjetil Klepper, S. Peter Klinken, Edda Kloppmann, Alan J. Knox, Yuichi Kodama, Yasushi Kogo, Miki Kojima, Soichi Kojima, Norio Komatsu, Hiromitsu Komiyama, Tsukasa Kono, Haruhiko Koseki, Shigeo Koyasu, Anton Kratz, Alexander Kukalev, Ivan Kulakovskiy, Anshul Kundaje, Hiroshi Kunikata, Richard Kuo, Tony Kuo, Shigehiro Kuraku, Vladimir A. Kuznetsov, Tae Jun Kwon, Matt Larouche, Timo Lassmann, Andy Law, Kim-Anh Le-Cao, Charles-Henri Lecellier, Weonju Lee, Boris Lenhard, Andreas Lennartsson, Kang Li, Ruohan Li, Berit Lilje, Leonard Lipovich, Marina Lizio, Gonzalo Lopez, Shigeyuki Magi, Gloria K. Mak, Vsevolod Makeev, Riichiro Manabe, Michiko Mandai, Jessica Mar, Kazuichi Maruyama, Taeko Maruyama, Elizabeth Mason, Anthony Mathelier, Hideo Matsuda, Yulia A. Medvedeva, Terrence F. Meehan, Niklas Mejhert, Alison Meynert, Norihisa Mikami, Akiko Minoda, Hisashi Miura, Yohei Miyagi, Atsushi Miyawaki, Yosuke Mizuno, Hiromasa Morikawa, Mitsuru Morimoto, Masaki Morioka, Soji Morishita, Kazuyo Moro, Efthymios Motakis, Hozumi Motohashi, Abdul Kadir Mukarram, Christine L. Mummery, Christopher J. Mungall, Yasuhiro Murakawa, Masami Muramatsu, Mitsuyoshi Murata, Kazunori Nagasaka, Takahide Nagase, Yutaka Nakachi, Fumio Nakahara, Kenta Nakai, Kumi Nakamura, Yasukazu Nakamura, Yukio Nakamura, Toru Nakazawa, Guy P. Nason, Chirag Nepal, Quan Hoang Nguyen, Lars K. Nielsen, Kohji Nishida, Koji M. Nishiguchi, Hiromi Nishiyori, Kazuhiro Nitta, Shuhei Noguchi, Shohei Noma, Cedric Notredame, Soichi Ogishima, Naganari Ohkura, Hiroshi Ohno, Mitsuhiro Ohshima, Takashi Ohtsu, Yukinori Okada, Mariko Okada-Hatakeyama, Yasushi Okazaki, Per Oksvold, Valerio Orlando, Ghim Sion Ow, Mumin Ozturk, Mikhail Pachkov, Triantafyllos Paparountas, Suraj P. Parihar, Sung-Joon Park, Giovanni Pascarella, Robert Passier, Helena Persson, Ingrid H. Philippens, Silvano Piazza, Charles Plessy, Ana Pombo, Fredrik Ponten, Stéphane Poulain, Thomas M. Poulsen, Swati Pradhan, Carolina Prezioso, Clare Pridans, Xiang-Yang Qin, John Quackenbush, Owen Rackham, Jordan Ramilowski, Timothy Ravasi, Michael Rehli, Sarah Rennie, Tiago Rito, Patrizia Rizzu, Christelle Robert, Marco Roos, Burkhard Rost, Filip Roudnicky, Riti Roy, Morten B. Rye, Oxana Sachenkova, Pal Saetrom, Hyonmi Sai, Shinji Saiki, Mitsue Saito, Akira Saito, Shimon Sakaguchi, Mizuho Sakai, Saori Sakaue, Asako Sakaue-Sawano, Albin Sandelin, Hiromi Sano, Yuzuru Sasamoto, Hiroki Sato, Alka Saxena, Hideyuki Saya, Andrea Schafferhans, Sebastian Schmeier, Christian Schmidl, Daniel Schmocker, Claudio Schneider, Marcus Schueler, Erik A. Schultes, Gundula Schulze-Tanzil, Colin A. Semple, Shigeto Seno, Wooseok Seo, Jun Sese, Jessica Severin, Guojun Sheng, Jiantao Shi, Yishai Shimoni, Jay W. Shin, Javier SimonSanchez, Asa Sivertsson, Evelina Sjostedt, Cilla Soderhall, Georges St Laurent, Marcus H. Stoiber, Daisuke Sugiyama, Kim M. Summers, Ana Maria Suzuki, Harukazu Suzuki, Kenji Suzuki, Mikiko Suzuki, Naoko Suzuki, Takahiro Suzuki, Douglas J. Swanson, Rolf K. Swoboda, Michihira Tagami, Ayumi Taguchi, Hazuki Takahashi, Masayo Takahashi, Kazuya Takamochi, Satoru Takeda, Yoichi Takenaka, Kin Tung Tam, Hiroshi Tanaka, Rica Tanaka, Yuji Tanaka, Dave Tang, Ichiro Taniuchi, Andrea Tanzer, Hiroshi Tarui, Martin S. Taylor, Aika Terada, Yasuhisa Terao, Alison C. Testa, Mark Thomas, Supat Thongjuea, Kentaro Tomii, Elena Torlai Triglia, Hiroo Toyoda, H. Gwen Tsang, Motokazu Tsujikawa, Mathias Uhlén, Eivind Valen, Marc van de Wetering, Erik van Nimwegen, Dmitry Velmeshev, Roberto Verardo, Morana Vitezic, Kristoffer Vitting-Seerup, Kalle von Feilitzen, Christian R. Voolstra, Ilya E. Vorontsov, Claes Wahlestedt, Wyeth W. Wasserman, Kazuhide Watanabe, Shoko Watanabe, Christine A. Wells, Louise N. Winteringham, Ernst Wolvetang, Haruka Yabukami, Ken Yagi, Takuji Yamada, Yoko Yamaguchi, Masayuki Yamamoto, Yasutomo Yamamoto, Yumiko Yamamoto, Yasunari Yamanaka, Kojiro Yano, Kayoko Yasuzawa, Yukiko Yatsuka, Masahiro Yo, Shunji Yokokura, Misako Yoneda, Emiko Yoshida, Yuki Yoshida, Masahito Yoshihara, Rachel Young, Robert S. Young, Nancy Y. Yu, Noriko Yumoto, Susan E. Zabierowski, Peter G. Zhang, Silvia Zucchelli, Martin Zwahlen, Clément Chatelain, Piero Carninci, Michiel J. L. de Hoon, Wyeth W. Wasserman, Laurent Bréhélin, Charles-Henri Lecellier

**Affiliations:** 1 Institut de Biologie Computationnelle, Montpellier, France; 2 Institut de Génétique Moléculaire de Montpellier, University of Montpellier, CNRS, Montpellier, France; 3 SANOFI R&D, Translational Sciences, Chilly Mazarin, France; 4 LIRMM, Univ Montpellier, CNRS, Montpellier, France; 5 RIKEN Center for Integrative Medical Sciences, Yokohama, Kanagawa Japan; 6 RIKEN Preventive Medicine and Diagnosis Innovation Program, Wako, Saitama Japan; 7 Artificial Intelligence Research Center, AIST, Tokyo, Japan; 8 Graduate School of Frontier Sciences, University of Tokyo, Chiba, Japan; 9 AIST-Waseda University CBBD-OIL, AIST, Tokyo, Japan; 10 Centre for Molecular Medicine and Therapeutics at the Child and Family Research Institute, Department of Medical Genetics, University of British Columbia, Vancouver, BC Canada; 11 Division of Genomic Technologies, RIKEN Center for Life Science Technologies, Yokohama, Japan; 12 MRC Human Genetics Unit, Institute of Genetics and Molecular Medicine, University of Edinburgh, Edinburgh, UK; 13 European Molecular Biology Laboratory, European Bioinformatics Institute, Wellcome Genome Campus, Hinxton, Cambridge UK; 14 Wellcome Trust Sanger Institute, Wellcome Trust Genome Campus, Hinxton, UK; 15 Computational Bioscience Research Centre, King Abdullah University of Science and Technology (KAUST), Thuwal, Saudi Arabia; 16 Department of Biochemistry, McGill University, Montreal, QC Canada; 17 UNSW Centre for Vascular Research, University of New South Wales, Sydney, NSW Australia; 18 Harry Perkins Institute of Medical Research, and the Centre for Medical Research, University of Western Australia, QEII Medical Centre, Perth, WA Australia; 19 Department of Systems Biology, Columbia University Medical Center, Columbia University, New York, NY USA; 20 The Bioinformatics Centre, Department of Biology, University of Copenhagen, Copenhagen, Denmark; 21 Biotech Research and Innovation Centre, University of Copenhagen, Copenhagen, Denmark; 22 RIKEN Omics Science Center (OSC), Yokohama, Japan; 23 Department of Transfusion Medicine and Stem Cell Regulation, Juntendo University Graduate School of Medicine, Tokyo, Japan; 24 Department of Statistics, University of California Berkeley, Berkeley, CA USA; 25 The Roslin Institute and Royal (Dick) School of Veterinary Studies, University of Edinburgh, Easter Bush, UK; 26 Department of Medicine, Karolinska Institute at Karolinska University Hospital, Huddinge, Sweden; 27 Artificial Intelligence Research Center (AIRC), National Institute of Advanced Industrial Science and Technology (AIST), Tokyo, Japan; 28 Biotechnology Research Institute for Drug Discovery, National Institute of Advanced Industrial Science and Technology (AIST), Tokyo, Japan; 29 Department of Dermatology and Allergy, Charit Campus Mitte, Universitatsmedizin Berlin, Berlin, Germany; 30 The Jackson Laboratory, Bar Harbor, ME USA; 31 Bioinformatics Institute, Agency for Science, Technology and Research (A*STAR), Singapore, Singapore; 32 Department of Computer Science, Stanford University, Stanford, CA USA; 33 Australian Institute for Bioengineering and Nanotechnology (AIBN), University of Queensland, Brisbane St Lucia, QLD Australia; 34 Department of Medical and Biological Sciences, University of Udine, Udine, Italy; 35 Cancer Science Institute of Singapore, National University of Singapore, Singapore, Singapore; 36 Department of Statistics, Oregon State University, Corvallis, OR USA; 37 McGill Centre for Bioinformatics and School of Computer Science, McGill University, Montral, QC Canada; 38 Genome Biology Unit, Istituto Nazionale di Genetica Molecolare (INGM) ‘Romeo and Enrica Invernizzi’, Milan, Italy; 39 Database Center for Life Science, Research Organization of Information and Systems, Tokyo, Japan; 40 Biozentrum, University of Basel, Basel, Switzerland; 41 Swiss Institute of Bioinformatics, Basel, Switzerland; 42 International Centre for Genetic Engineering and Biotechnology, Cape Town Component, Cape Town, South Africa; 43 Division of Immunology, Institute of Infectious Diseases and Molecular Medicine, Health Science Faculty, University of Cape Town, Cape Town, South Africa; 44 Genomics Division, Lawrence Berkeley National Laboratory, Berkeley, CA USA; 45 National Center for Biotechnology Information, National Library of Medicine, National Institutes of Health, Bethesda, MD USA; 46 Berlin Institute for Medical Systems Biology, Max-Delbruck Centre for Molecular Medicine, Berlin, Germany; 47 Biotechnology Center, Technische Universitat Dresden, Dresden, Germany; 48 Sorbonne Universités, Université Pierre et Marie Curie, Laboratoire de Biologie Computationnelle et Quantitative, Paris, France; 49 Telethon Kids Institute, The University of Western Australia, Subiaco, WA Australia; 50 Department of Medical Genetics, Centre for Molecular Medicine and Therapeutics, Child and Family Research Institute, University of British Columbia, Vancouver, BC Canada; 51 Graduate Program in Bioinformatics, University of British Columbia, Vancouver, BC Canada; 52 Fondazione Bruno Kessler, Trento, Italy; 53 Children’s Hospital at Westmead, Sydney, NSW Australia; 54 Laboratorio Nazionale Consorzio Italiano Biotecnologie (LNCIB), Trieste, Italy; 55 Department of Gastroenterology, Medical Section, Herlev Hospital, University of Copenhagen, Herlev, Denmark; 56 Functional Genomics, Cold Spring Harbor Laboratory, Cold Spring Harbor, NY USA; 57 Centre for Integrative Bioinformatics (IBIVU), VU University Amsterdam, Amsterdam, The Netherlands; 58 Institute of Natural and Mathematical Sciences, Massey University Auckland, Albany, New Zealand; 59 Ecole Polytechnique Fdrale de Lausanne and Swiss Institute of Bioinformatics, Lausanne, Switzerland; 60 Institute of Pharmaceutical Sciences, Swiss Federal Institute of Technology, ETH Zurich, Zurich, Switzerland; 61 Institute of Fundamental Medicine and Biology, Kazan Federal University, Kazan, Russia; 62 Telethon Institute of Genetics and Medicine (TIGEM), Pozzuoli, Italy; 63 Department of Neurology, University at Buffalo School of Medicine and Biomedical Sciences, Buffalo, NY USA; 64 Department of Biostatistics, Harvard School of Public Health, Boston, MA USA; 65 Centre for Genomic Regulation (CRG), The Barcelona Institute of Science and Technology, Barcelona, Spain; 66 Department of Gastroenterology, Research Center for Hepatitis and Immunology, Research Institute, National Center for Global Health and Medicine, Chiba, Japan; 67 Department of Cancer Research and Molecular Medicine, Norwegian University of Science and Technology, Trondheim, Norway; 68 Department of Otology and Laryngology, Harvard Medical School, Boston, MA USA; 69 Department of Internal Medicine III, University Hospital Regensburg, Regensburg, Germany; 70 Regensburg Centre for Interventional Immunology (RCI), Regensburg, Germany; 71 Department of Biosciences and Nutrition, Karolinska Institute, Stockholm, Sweden; 72 Department of Biochemistry and Biophysics, Stockholm University, Stockholm, Sweden; 73 Division of Neural Differentiation and Regeneration, Kobe University Graduate School of Medicine, Kobe, Japan; 74 Department of Psychiatry and Behavioral Sciences, University of Miami Miller School of Medicine, Miami, FL USA; 75 F.M. Kirby Neurobiology Center, Department of Neurology, Boston Children’s Hospital, Harvard Medical School, Boston, MA USA; 76 Department of Biological Sciences, University of Delaware, Newark, DE USA; 77 Department of Biochemistry and Cell Biology, Rice University, Houston, TX USA; 78 Department of Bioengineering, Rice University, Houston, TX USA; 79 Mater Research Institute, and Queensland Brain Institute, University of Queensland, Brisbane, QLD Australia; 80 Vavilov Institute of General Genetics, Russian Academy of Sciences, Moscow, Russia; 81 Department of Oncology, Division of Biostatistics and Bioinformatics, Johns Hopkins University School of Medicine, Baltimore, MD USA; 82 Genome Function Group, MRC Clinical Sciences Centre, Imperial College London, London, UK; 83 Science for Life Laboratory, KTH-Royal Institute of Technology, Stockholm, Sweden; 84 Department of Computational Biology and Medical Sciences, University of Tokyo, Tokyo, Japan; 85 Research Institute for Diseases of Old Age, Juntendo University Graduate School of Medicine, Tokyo, Japan; 86 RIKEN Quantitative Biology Center, Suita, Japan; 87 Graduate School of Information Science and Technology, Osaka University, Suita, Japan; 88 Department of Biomedicine, Bioinformatics Core Facility, University Hospital Basel, Basel, Switzerland; 89 Academic Medical Center, University of Amsterdam, Amsterdam, The Netherlands; 90 The Systems Biology Institute, Tokyo, Japan; 91 Division of Biological and Environmental Sciences & Engineering, King Abdullah University of Science and Technology (KAUST), Thuwal, Saudi Arabia; 92 Department for Bioinformatics and Computational Biology, Technische UniversitŁt Mnchen, Garching, Germany; 93 Department of Computer Science, University of Bristol, Bristol, UK; 94 Institute of Biotechnology, University of Helsinki, Helsinki, Finland; 95 Area of Neuroscience, International School for Advanced Studies (SISSA), Trieste, Italy; 96 Department of Neuroscience and Brain Technologies, Italian Institute of Technologies (IIT), Genoa, Italy; 97 Faculty of Medicine, Imperial College London, London, UK; 98 Department of Biology, University of Bergen, Bergen, Norway; 99 Department of Proteomics, KTH-Royal Institute of Technology, Stockholm, Sweden; 100 Department of Electrical Engineering and Bioscience, Faculty of Science and Engineering, Waseda University, Tokyo, Japan; 101 RIKEN Center for Life Science Technologies, Division of Bio-Function Dynamics Imaging, Kobe, Japan; 102 Department of Neurology, Juntendo University Graduate School of Medicine, Tokyo, Japan; 103 Department of Treatment and Research in Multiple Sclerosis and Neuro-intractable Disease, Juntendo University Graduate School of Medicine, Tokyo, Japan; 104 Department of Research for Parkinsons Disease, Juntendo University Graduate School of Medicine, Tokyo, Japan; 105 Department of Stem Cells and Applied Medicine, Osaka University Graduate School of Medicine, Suita, Japan; 106 Department of Ophthalmology, Osaka University Graduate School of Medicine, Suita, Japan; 107 Melanoma Research Center, The Wistar Institute, Philadelphia, PA USA; 108 German Center for Neurodegenerative Diseases (DZNE), Tubingen, Germany; 109 Sheffield Institute for Translational Neuroscience, University of Sheffield, Sheffield, UK; 110 Australian Infectious Diseases Research Centre (AID), University of Queensland, Brisbane, QLD Australia; 111 Department of Human Genetics, Leiden University Medical Center, Leiden, The Netherlands; 112 Department of Respiratory Medicine, Graduate School of Medicine, University of Tokyo, Tokyo, Japan; 113 Molecular Profiling Research Center for Drug Discovery, National Institute of Advanced Industrial Science and Technology (AIST), Tokyo, Japan; 114 Computational Biology Research Center, National Institute of Advanced Industrial Science and Technology (AIST), Tokyo, Japan; 115 The University of Melbourne Centre for Stem Cell Systems, School of Biomedical Sciences, The University of Melbourne, Melbourne, VIC Australia; 116 Walter and Eliza Hall Institute of Medical Research, Melbourne, VIC Australia; 117 RIKEN Bioinformatics and Systems Engineering Division (BASE), Yokohama, Japan; 118 Medical Research Support Center, Kyoto University Graduate School of Medicine, Kyoto, Japan; 119 Department of Bioscience, Nagahama Institute of Bio-Science and Technology, Nagahama, Japan; 120 Center for Information Biology and DNA Data Bank of Japan, National Institute of Genetics, Mishima, Japan; 121 Laboratory Animal Research Center, Institute of Medical Science, University of Tokyo, Tokyo, Japan; 122 Department of Obstetrics and Gynecology, Juntendo University, Tokyo, Japan; 123 Institute of Genomics, School of Biomedical Sciences, Huaqiao University, Xiamen, China; 124 St. Laurent Institute, Woburn, MA USA; 125 A.N. Belozersky Institute of Physico-Chemical Biology, Lomonosov Moscow State University, Moscow, Russia; 126 Department of Ophthalmology, Kyoto Prefectural University of Medicine, Kyoto, Japan; 127 Diamantina Institute, University of Queensland, Brisbane St Lucia, QLD Australia; 128 Folkhalsan Institute of Genetics, Helsinki, Finland; 129 Science for Life Laboratory, Karolinska Institute, Solna, Sweden; 130 Department of Computational Biology, Faculty of Frontier Sciences, University of Tokyo, Chiba, Japan; 131 RIKEN Center for Developmental Biology, Kobe, Japan; 132 Division of Cellular Therapy, Institute of Medical Science, University of Tokyo, Tokyo, Japan; 133 Division of Stem Cell Signaling, Institute of Medical Science, University of Tokyo, Tokyo, Japan; 134 Sony Computer Science Laboratories, Inc, Tokyo, Japan; 135 Systems Biology Institute (SBI) Australia, Monash University, Clayton, VIC Australia; 136 Okinawa Institute of Science and Technology, Onna, Japan; 137 Department of Respiratory Medicine and Nottingham Respiratory Research Unit, University of Nottingham, Nottingham, UK; 138 Department of Hematology, Juntendo University Graduate School of Medicine, Tokyo, Japan; 139 Department of Coloproctological Surgery, Faculty of Medicine, Juntendo University School of Medicine, Tokyo, Japan; 140 Department of Microbiology and Immunology, Keio University School of Medicine, Tokyo, Japan; 141 Engelhardt Institute of Molecular Biology, Russian Academy of Sciences, Moscow, Russia; 142 Skolkovo Institute of Science and Technology, Moscow, Russia; 143 Department of Genetics, Stanford University, Stanford, CA USA; 144 Department of Ophthalmology and Visual Science, Tohoku University Graduate School of Medicine, Sendai, Japan; 145 Department of Retinal Disease Control, Tohoku University Graduate School of Medicine, Sendai, Japan; 146 Institute of Molecular Genetics of Montpellier, Montpellier, France; 147 Department of Dermatology, Kyungpook National University School of Medicine, Daegu, South Korea; 148 Department of Mathematical Sciences, University of Copenhagen, Copenhagen, Denmark; 149 Center for Molecular Medicine and Genetics, Wayne State University, Detroit, MI USA; 150 Department of Neurology, School of Medicine, Wayne State University, Detroit, MI USA; 151 Department of Medical and Biological Physics, Moscow Institute of Physics and Technology, Moscow, Russia; 152 Department of Systems and Computational Biology, Albert Einstein College of Medicine, New York, NY USA; 153 IMPPC, Institute of Predictive and Personalized Medicine of Cancer, Badalona, Spain; 154 Institute of Bioengineering, Research Center of Biotechnology, Moscow, Russia; 155 Immunology Frontier Research Center, Osaka University, Suita, Japan; 156 Kanagawa Cancer Center Research Institute, Yokohama, Japan; 157 RIKEN Brain Science Institute, Saitama, Japan; 158 Research Center for Genomic Medicine, Saitama Medical University, Saitama, Japan; 159 Department of Medical Life Science, Graduate School of Medical Life Science, Yokohama City University, Yokohama, Japan; 160 Department of Gene Expression Regulation, Institute of Development, Aging and Cancer, Tohoku University, Sendai, Japan; 161 Department of Anatomy and Embryology, Leiden University Medical Center, Leiden, The Netherlands; 162 Department of Obstetrics and Gynecology, Graduate School of Medicine, University of Tokyo, Tokyo, Japan; 163 Human Genome Center, The Institute of Medical Science, University of Tokyo, Tokyo, Japan; 164 RIKEN BioResource Center, Tsukuba, Japan; 165 Department of Advanced Ophthalmic Medicine, Tohoku University Graduate School of Medicine, Sendai, Japan; 166 School of Mathematics, University of Bristol, Bristol, UK; 167 Department of Informatics, University of Bergen, Bergen, Norway; 168 Tohoku Medical Megabank Organization, Tohoku University, Sendai, Japan; 169 Department of Frontier Research in Tumor Immunology, Center of Medical Innovation and Translational Research, Osaka University, Osaka, Japan; 170 Department of Biochemistry, Ohu University School of Pharmaceutical Sciences, Koriyama, Japan; 171 Department of Statistical Genetics, Osaka University Graduate School of Medicine, Suita, Japan; 172 Institute for Protein Research, Osaka University, Suita, Japan; 173 Dulbecco Telethon Institute at IRCSS Fondazione Santa Lucia, Rome, Italy; 174 Division of Oncology and Pathology, Department of Clinical Sciences, Lund University, Lund, Sweden; 175 Department of Immunobiology, Biomedical Primate Research Centre, Rijswijk, The Netherlands; 176 Department of Immunology, Genetics and Pathology, Uppsala University, Uppsala, Sweden; 177 Science for Life Laboratory, Uppsala University, Uppsala, Sweden; 178 Department of BioSciences, Rice University, Houston, TX USA; 179 Center for Translational Cancer Research, Helen F. Graham Cancer Center & Research Institute, Newark, DE USA; 180 Department of Biomedical Engineering, University of Delaware, Newark, DE USA; 181 Department of Biostatistics and Computational Biology, Dana-Farber Cancer Institute and Harvard Medical School, Boston, MA USA; 182 Department of Biostatistics, Harvard T.H. Chan School of Public Health, Boston, MA USA; 183 Program in Cardiovascular and Metabolic Disorders, DukeNUS Medical School, Singapore, Singapore; 184 Department of Computer and Information Science, Norwegian University of Science and Technology, Trondheim, Norway; 185 Division of Breast Oncology, Juntendo University School of Medicine, Tokyo, Japan; 186 Division for Health Service Promotion, University of Tokyo, Tokyo, Japan; 187 Department of Experimental Pathology, Institute for Frontier Medical Sciences, Kyoto University, Kyoto, Japan; 188 Department of Allergy and Rheumatology, Graduate School of Medicine, University of Tokyo, Tokyo, Japan; 189 Biomedical Research Centre at Guy’s and St Thomas’ Trust, Genomics Core Facility, Guy’s Hospital, London, UK; 190 Division of Gene Regulation, Institute for Advanced Medical Research, Keio University School of Medicine, Tokyo, Japan; 191 Department of Informatics, Technische UniversitŁt Mnchen, Garching, Germany; 192 Paracelsus Medical University, Institute of Anatomy, Nuremberg, Germany; 193 Department of Computer Science, Tokyo Institute of Technology, Tokyo, Japan; 194 International Research Center for Medical Sciences, Kumamoto University, Kumamoto, Japan; 195 Department of Neurology and Center for Translational Systems Biology, Mount Sinai School of Medicine, New York, NY USA; 196 Department of Molecular Biology, Cell Biology, and Biochemistry, Brown University, Providence, RI USA; 197 Department of Research and Development of Next Generation Medicine, Faculty of Medical Sciences, Kyushu University, Fukuoka, Japan; 198 Department of General Thoracic Surgery, Juntendo University School of Medicine, Tokyo, Japan; 199 Center for Radioisotope Sciences, Tohoku University Graduate School of Medicine, Sendai, Japan; 200 Department of Systems Biology, Graduate School of Biochemical Science, Tokyo Medical and Dental University, Tokyo, Japan; 201 Department of Plastic and Reconstructive Surgery, Juntendo University Graduate School of Medicine, Tokyo, Japan; 202 RIKEN Advanced Center for Computing and Communication, Preventive Medicine and Applied Genomics Unit, Yokohama, Japan; 203 Department of Clinical Molecular Genetics, School of Pharmacy, Tokyo University of Pharmacy and Life Sciences, Tokyo, Japan; 204 Hubrecht Institute, Utrecht, The Netherlands; 205 Department of Biological Information, Graduate School of Bioscience and Biotechnology, Tokyo Institute of Technology, Tokyo, Japan; 206 Department of Biochemistry, Nihon University School of Dentistry, Tokyo, Japan; 207 Graduate School of Medicine, Tohoku University, Sendai, Japan; 208 Faculty of Information Science and Technology, Osaka Institute of Technology, Hirakata, Japan; 209 The SKI Stem Cell Research Facility, The Center for Stem Cell Biology and Developmental Biology Program, Sloan Kettering Institute, New York, NY USA; 210 Department of Health Sciences, Universit del Piemonte Orientale, Novara, Italy

**Keywords:** Machine learning, Genomics, Transcriptomics

Correction to: *Nature Communications* 10.1038/s41467-021-23143-7, published online 2 June 2021.

The original version of this Article incorrectly included Kristina Hettne as a current member of the FANTOM consortium at the time of publication. This has now been corrected in both the PDF and HTML versions of the Article.

