# Supplementary material for: Discovery of widespread transcription initiation at microsatellites predictable by sequence-based deep neural network
Source: Nat Commun. 2021 Jun 2;12:3297. doi: 10.1038/s41467-021-23143-7 (PMC8172540; doi:10.1038/s41467-021-23143-7)
Supplement: Supplementary file 1 — Supplementary Information [file 41467_2021_23143_MOESM1_ESM.pdf]

Supplementary Methods: Cap Trap and MinION sequencing

1. Addition of polyA tail

The polyA tail addition was carried out by using 8 ug of totalRNA in 14.5ul of water, 2.0ul of 10x PolyA polymerase buffer (NEB), 2.0ul of 10mM ATP (NEB), 1.0ul of RNaseOUT (Invitrogen) and 0.5ul of PolyA polymeras(5 U/ul). We incubated this reaction mix at 37C for 15m, then put the tube on ice. After polyA polymerase reaction, polyA-tailed totalRNA (PAPed RNA) was purified with Agencourt RNAClean XP kit (Beckman coulter) according to the manufacturer' s instructions and eluted in 40ul of water.

2. Reverse Transcription

We put 5ul each of PAPed RNA into 8 wells.

The cDNA synthesis was carried out by using 5ug of total RNA or 1ug of PAPed RNA in 5ul of water and 0.5ul of 100uM RT primer (5' - TTTTTTTTUUUTTTTTVN -3') by PrimeScript II Reverse Transcriptase(TaKaRa). We heated RNA and primer at 65C for 5min and then placed them on ice. Then we added the reaction mixture, 4ul of 5x PrimeScript II buffer, 4ul of water, 1ul of RNaseOUT and 1ul of PrimeScript II, followed by reverse transcription in a thermal cycler: 42C for 60min, then chilled at 4C.

After the reaction, the cDNA/RNA hybrids were purified with Agencourt RNAClean XP.

3. Oxidation / Biotinylation

To oxidize the diol residue of Cap structure, 40ul of purified cDNA/RNA hybrids were mixed with 2ul of 1M NaOAc (pH4.5) and 2ul of 250mM $NaIO_4$ (Sigma-Aldrich) and incubated on ice for 5min in dark. To stop the reaction, the oxidized cDNA/RNA hybrids were mixed with 16ul of 1M Tris-HCl(pH8.5). The sample was purified with RNAClean XP. Four ul of 1M NaOAc (pH6.2) and 4ul of 100mM Biotin (long arm) hydrazide (Vector Laboratories) in DMSO were added and the reaction mixture were incubated at 40C for 30min. After the incubation, the biotinylated sample was purified with RNAClean XP. Finally, single-strand RNA regions which were not protected by a complementary first-strand cDNA strand were digested using RNaseONE(Promega) by addition of 4.5ul of 10×RNaseI buffer and 0.5ul of RNaseONE and incubation at 37C for 30min. The reaction mixture were purified with RNAClean XP.

4. CapTrap

Thirty microliters of Dynabeads M-270 Streptavidin beads slurry (ThermoFisher Scientific) was washed with 30ul of LiCl binding buffer (7M LiCl, 10mM Tris-HCl (pH7.5), 0.1% Tween20, 2mM EDTA (pH8.0)) twice and resuspended in 95ul of LiCl buffer. The washed M-270 beads were added to 40ul of purified biotinylated cDNA/RNA hybrids. Binding was carried out for 15min at 37C, then beads were purified using a magnetic bar and washed with TE wash buffer (10mM Tris-HCl (pH7.5), 0.1% Tween20, 1mM EDTA(pH8.0)) three times.

Captured cDNA was released from the beads by heat shock and RNaseI treatment. Beads were resuspended in 35ul of release buffer (1x RNaseONE buffer, 0.01% Tween20), incubated at 95C for 5min and chilled on ice immediately. The supernatant containing cDNA was transferred to a new tube. The beads were washed with 30ul of release buffer, and the supernatant was pooled together with the first elution. Then the sample was treated with RNase (0.1ul of 60U/ul RNaseH (TaKaRa) and 2ul of 10U/ul RNaseI for 30min at 37C) to remove RNA completely. Then the Cap-Trapped cDNA was purified with Agencourt AMPure XP (Beckman coulter) according to the manufacture’s protocol. The cDNA quantity was determined with the Quant-iT OliGreen ssDNA Assay kit (ThermoFisher Scientific).

5. Linker Ligation

5’/3’ linkers was ligated to the both end of Cap-trapped cDNA.

5.1 How to make a linker

Dissolve the oligonucleotides of 5’ linker to 1mM in TE buffer. For the annealing reaction, GN5 linker reaction solution (4ul of 5’ linker up GN5 (5’-GTGGTAUCAACGCAGAGUACGNNNNN -P-3' : 1mM), 4ul of 5’ linker down (5'-P-GTACTCTGCGTTGATACCAC-P-3' : 1mM), 4 ul of 1M NaCl and 28 ul of water) and N6 linker reaction solution (1ul of 5’ linker up N6 (5'-GTGGTAUCAACGCAGAGUACNNNNNN -P-3' : 1mM), 1ul of 5’ linker down (1mM), 1 ul of 1M NaCl and 7 ul of water) were incubated the following conditions: 95°C, 5 min gradient 0.1°C/sec, 83°C, 5 min, gradient 0.1°C/sec, 71°C 5 min, gradient 0.1°C/sec, 59°C 5 min, gradient 0.1°C/sec, 59°C 5 min, gradient 0.1°C/sec, 47°C 5 min, gradient 0.1°C/sec, 35°C 5 min, gradient 0.1°C/sec, 23°C 5 min, gradient 0.1°C/sec and 11°C Hold. The annealed GN5 linker solution(40ul) and N6 linker solution(10ul) were mixed

(5’CTR-Seq linker (100uM)). The 5’CTR-Seq linker (100uM) was diluted to 10uM with 0.1M NaCl (in TE).

For the 3’CTR-Seq linker, 1ul of 3’CTR-Seq up (5'-AAAAABBBBBBBBGCAUCGCUGTCTCUTAUACACAUCUCCGAGCCCACGAGAC-P-3') and 1ul of 3’CTR-Seq down (5'-GTCTCGTGGGCTCGGAGATGTGTATAAGAGACAGCGATGC -3'), 1 ul of 1M NaCl and 7 ul of wather. Then incubate the mixed solution as same condition as the 5’linker. After annealing step, the UMI part (BBBBBBBB) was filled with Phusion High-Fidelity DNA polymerase (NEB) and dVTPs(dATP/dGTP/dCTP) instead of dNTPs. After filling reaction, the 3’linker solution was purified with AMPure XP. Then adjust the concentration to 10uM with 0.1M NaCl in TE buffer.

5.2 5’SSLL

The cDNA solution was dried up using a SpeedVac (80C for 35min). The pellet was dissolved in 4ul of water. After incubation of cDNA solution at 95C for 5min and chilled on ice for 2min, 1ul of 5’CTR-Seq linker (10uM), which was incubated at 55C for 5min and chilled on ice, was added. Then 10ul of Mighty Mix (TaKaRa) was added, mixed gently and incubated at 30C for 4h. The sample after ligation was purified with AMPure XP.

5.3 USER

To shorten the long polyT stretch of RT primer, the U residues in the RT primer were digested with USER enzyme (NEB). We added 2ul of USER enzyme (2U/ul), 5ul of 10x CutSmart buffer (NEB) and 3ul of water to 40ul of 5’linker ligated cDNA. We incubated the reaction solution at 37C for 30min and chilled on ice.

Then the dT stretch at 5’end of cDNA became 5nt. The cDNA was purified with AMPure XP beads.

5.4 3’SSLL

The cDNA solution was dried up using a SpeedVac (80C for 35min). The pellet was dissolved in 4ul of water. After incubation of cDNA solution at 95C for 5min and chilled on ice for 2min, 1ul of 3’CTR-Seq linker (10uM), which was incubated at 65C for 5min and chilled on ice, was added. Then 10ul of Mighty Mix was added, mixed gently and incubated at 16C for 16h. The sample after ligation was purified with AMPure XP.

6. SAP treatment

To digest excessed 3' linker and dephosphorylate the 3'end of 5'linker down strand, the cDNA was treated with 1ul of SAP (Affymetrics) and 2ul of USER in 1x SAP buffer, incubated at 37C for 30min. After reaction, the cDNA was purified with AMPure XP.

7. 2nd strand synthesis

The cDNA solution was concentrated to 5ul using a SpeedVac (80C for 35min). The 2nd strand synthesis was carried out using 5ul of cDNA, 0.5ul of 2nd primer (5' - TCGTCGGCAGCGTCAGATGTGTATAAGAGACAGNNNNNNNNGTGGTATCAACGCAGAGTAC -3' :100uM), 1.3ul of DMSO, 5.8ul of water and 12.5ul of 2x KAPA HiFi HS mix (NIPPON Genetics). The reaction mix was incubated for the following condition; 95C for 5min, 55C for 5min, 72C for 30min and hold at 4C. After 2nd strand synthesis, the excessed primer were digested with adding 1ul of Exonuclease I (20U/ul, NEB) and incubation at 37C for 30min. Then the sample solution was purified with AMPure XP twice. The volume of used AMPure XP beads was 46.8ul at 1st purification and 40ul at 2nd purification. The sample was dried up with SpeedVac (37C for 75min). The pellet was dissolved in 7ul of water.

8. quantification/qualification

The ds cDNA was quantified using Quant-iT PicoGreen Assay kit (Thermofisher Scientific), according to the manufacturer' s instructions. For quantification, we used 1ul of ds-cDNA. And we analyzed the length distribution with Agilent High Sensitivity DNA kit (Agilent).

9. cDNA amplification

The double stranded cDNAs were amplified using Illumina adapter-specific primers and LongAmp Taq DNA polymerase (NEB). After 16 cycles of PCR (8 minutes for elongation time), amplified cDNAs were purified with equal volume of AMPure XP beads (Beckmann Coulter).

10. NanoPore Sequencing

Purified cDNAs were subjected to Nanopore sequencing library following to manufacturer's 1D ligation sequencing protocol (version NBE_9006_v103_revO_21Dec2016). Nanopore libraries were sequenced by MinION Mk1b with R9.4 flowcell. Sequence data was generated by MinKNOW 1.7.14

11. NanoPore Basecalling

In order to generate fastq files from FAST5 files, Basecalling was processed by "Albacore v2.1.0" basecaller software which was provided from Oxford NanoPore Technologies.

12. Trimming adapter sequence from fastq file

To preparing clean reads from fastq files, trimming was processed by "Porechop v0.2.3".

13. Method for aligning RIKEN MinION cDNA reads to the human genome

* Software versions: LAST 941, Python 2

First, an index (named "hdb") of the genome and linkers was prepared:

```
lastdb -P0 -uNEAR -R01 hdb hg38.analysisSet.fa linkers.fa
```

Then, the rates of insertion, deletion, and substitution between reads and genome were estimated:

```
last-train -P0 --matsym hdb BC01_A549_OligoDT.fa > f6nano.mat
```

This was done for BC01 and BC02, with and without --matsym. The results were similar, and the result of the above command was used in the next steps.

The reads were aligned to the linkers:

```
lastdb -c -uNEAR linkerdb linkers.fa

echo "N 0 0 0 0" | cat f6nano.mat - |
lastal -P0 -p- linkerdb reads.fa | last-split -m1 > linkers-reads.maf
```

(This adds a row of zero scores for N to the score matrix, which is appropriate for the UMI with Ns. The other UMI with Vs/Bs is scored appropriately by default.)

Then the reads were oriented in the RNA forward-strand direction:

```
analyze-linkers.py reads.fa linkers-reads.maf > reads-fwd.fa 2> linkers-reads.txt
```

The .txt files have some statistics on linker analysis failures.

Finally, the reads were aligned to the genome:

```
parallel-fasta -k "lastal -p f6nano.mat -d90 -m50 -D10 hdb | last-split -g hdb -m1" < reads-fwd.fa > reads.maf
```

And alternative alignment formats were prepared:

```
maf-convert -j1e6 psl reads.maf | grep -v linker > reads.psl
pslToBed reads.psl reads.bed
```

Warnings

* The results include low-confidence alignments. In the maf files, each alignment has a "mismap" probability, which is the estimated probability that it's aligned to the wrong place.

* There are probably some incorrect alignments to processed pseudogenes. It's hard to avoid these completely. (There may also be correct alignments to processed pseudogenes.)

* There may be an artifactual tendency for first exons to begin just after AG, and last exons to end just before GT. This is because the spliced alignment method does not treat linkers differently.

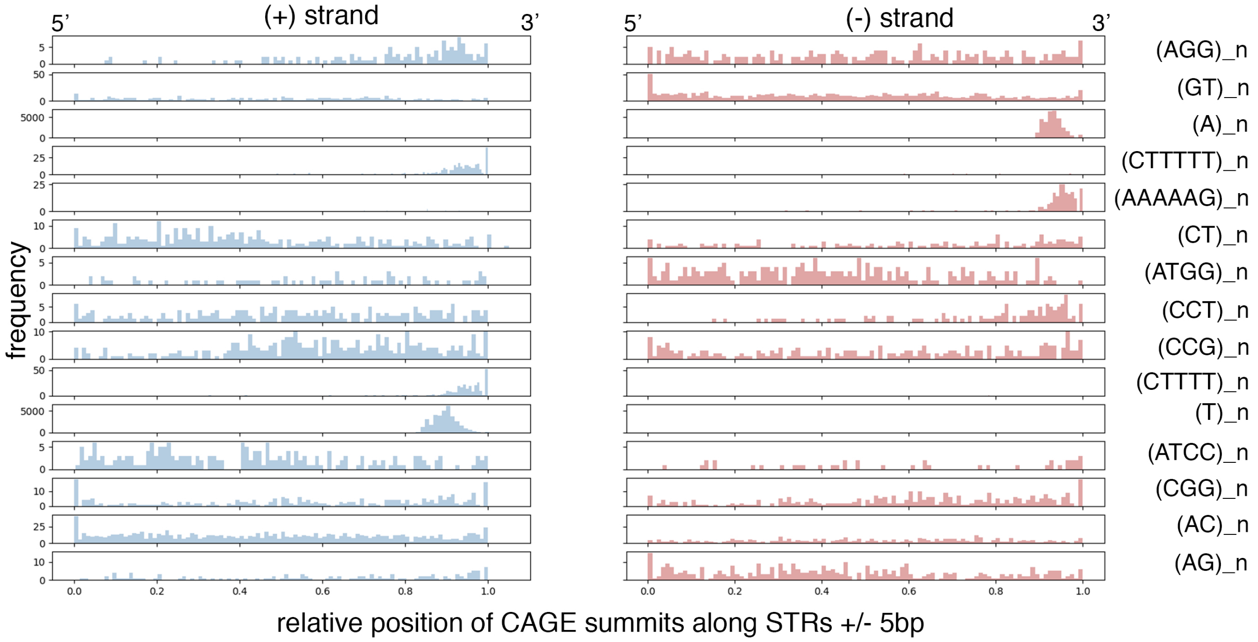

Supplementary Figure 1 – **Distribution of CAGE peak summits along STRs.** x-axis, the relative position was computed on a window corresponding to STR length ± 5bp ; y-axis, frequency of CAGE peaks. Only STR classes with > 200 CAGE peaks on (+) strand and > 200 CAGE peaks on (-) strands are shown.

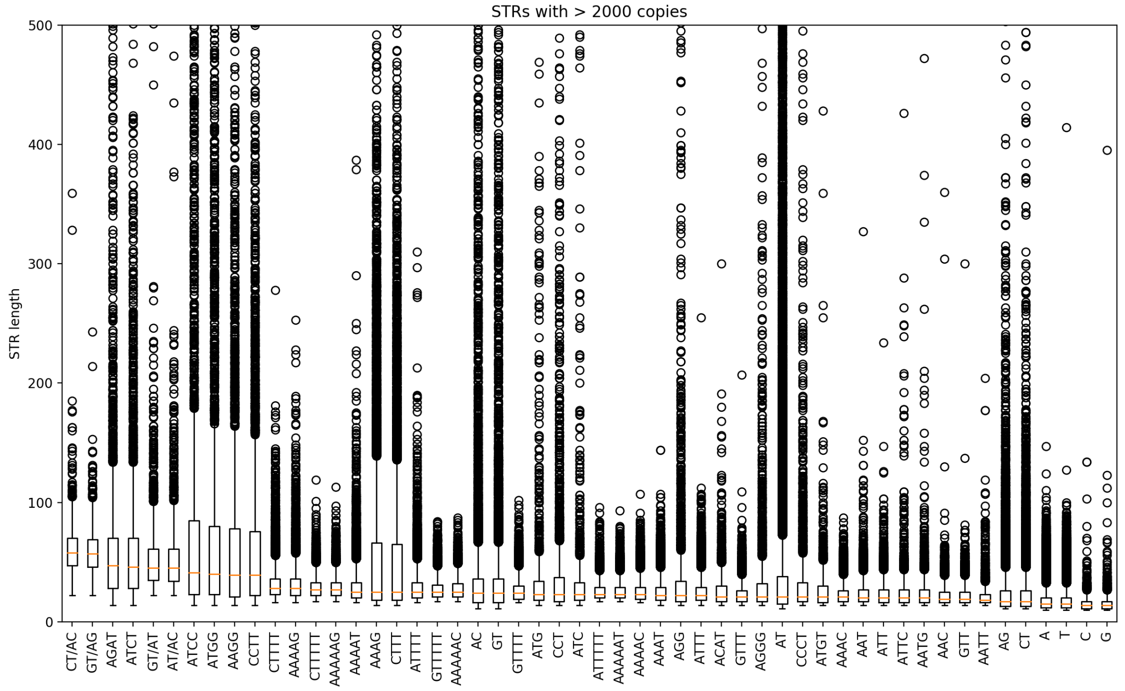

Supplementary Figure 2 – **STR length distribution in different classes.** STR classes are sorted by median length. Only STR classes with > 2,000 elements are shown. Boxplots are defined as in Figure 1d.

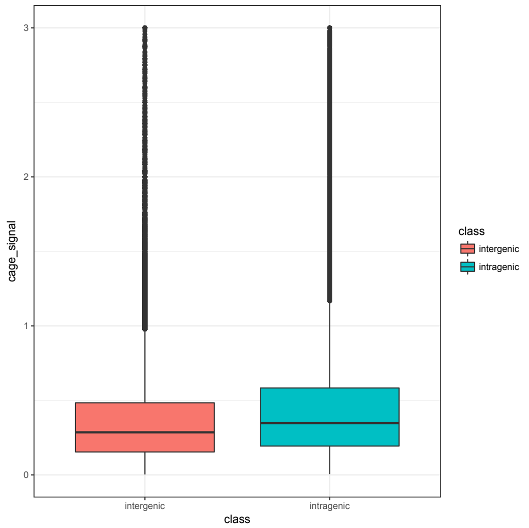

Supplementary Figure 3 – **CAGE signal at inter- and intragenic STRs.** FANTOM CAT annotation [1] was used to define inter- and intragenic STRs. 1,195,065 out of 1,620,030 STRs from the HipSTR catalog, which is only defined on the (+) strand, are located within FANTOM CAT genes, no matter their strand. It is however possible to evaluate the CAGE signal on both strands of an STR (see Methods section). CAGE signal (y-axis) computed for intragenic STRs (blue) corresponds to the signal measured at STRs located within, and in the same orientation of, one FANTOM CAT gene (n = 1,309,455) (option -*s* of bedtools *intersect*). Conversely, CAGE signal computed for intergenic STRs (red) corresponds to the signal measured at STRs located outside FANTOM CAT genes (n = 1,766,779) (option -*v* of bedtools *intersect*). Boxplots are defined as in Figure 1d. Median CAGE signal for intragenic STRs = 0.57143 ; median CAGE signal for intergenic STRs = 0.52174 (two-sided Wilcoxon test p-value $< 2.2e\text{-}16$). The statistical significance of the test is merely due to the high number of elements considered in each case. By reducing that number to, for instance, 500 randomly chosen elements, this p-value can increase to 0.1544. We therefore concluded that there is no drastic difference between the CAGE signals observed at intra- and intergenic STRs.

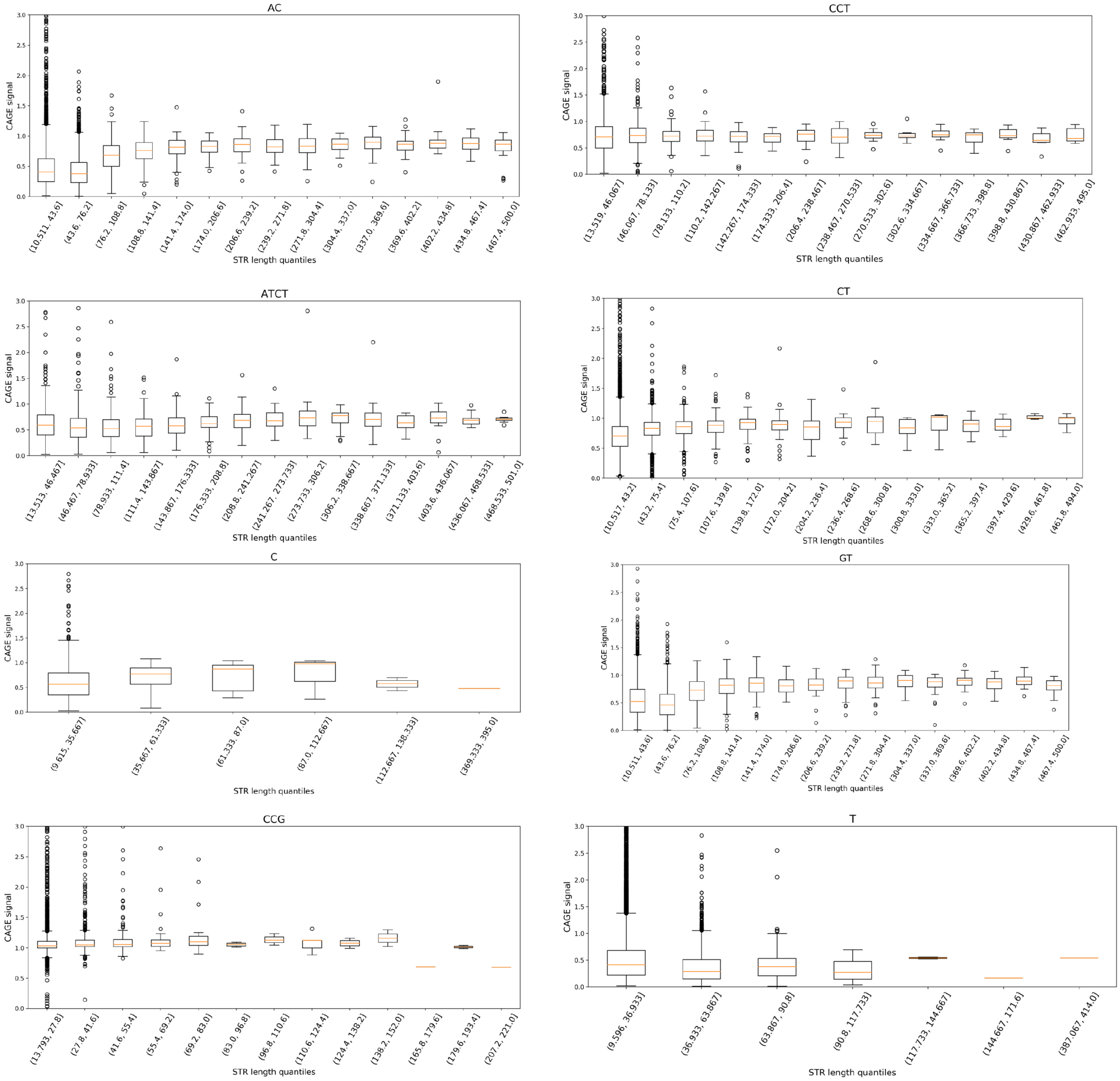

Supplementary Figure 4 – **CAGE signal in different STR classes according to STR length.** Quantiles were defined using the Pandas quantile-based discretization *qcut* function. x-axis: quantiles ; y-axis: CAGE signal. Boxplots are defined as in Figure 1d.

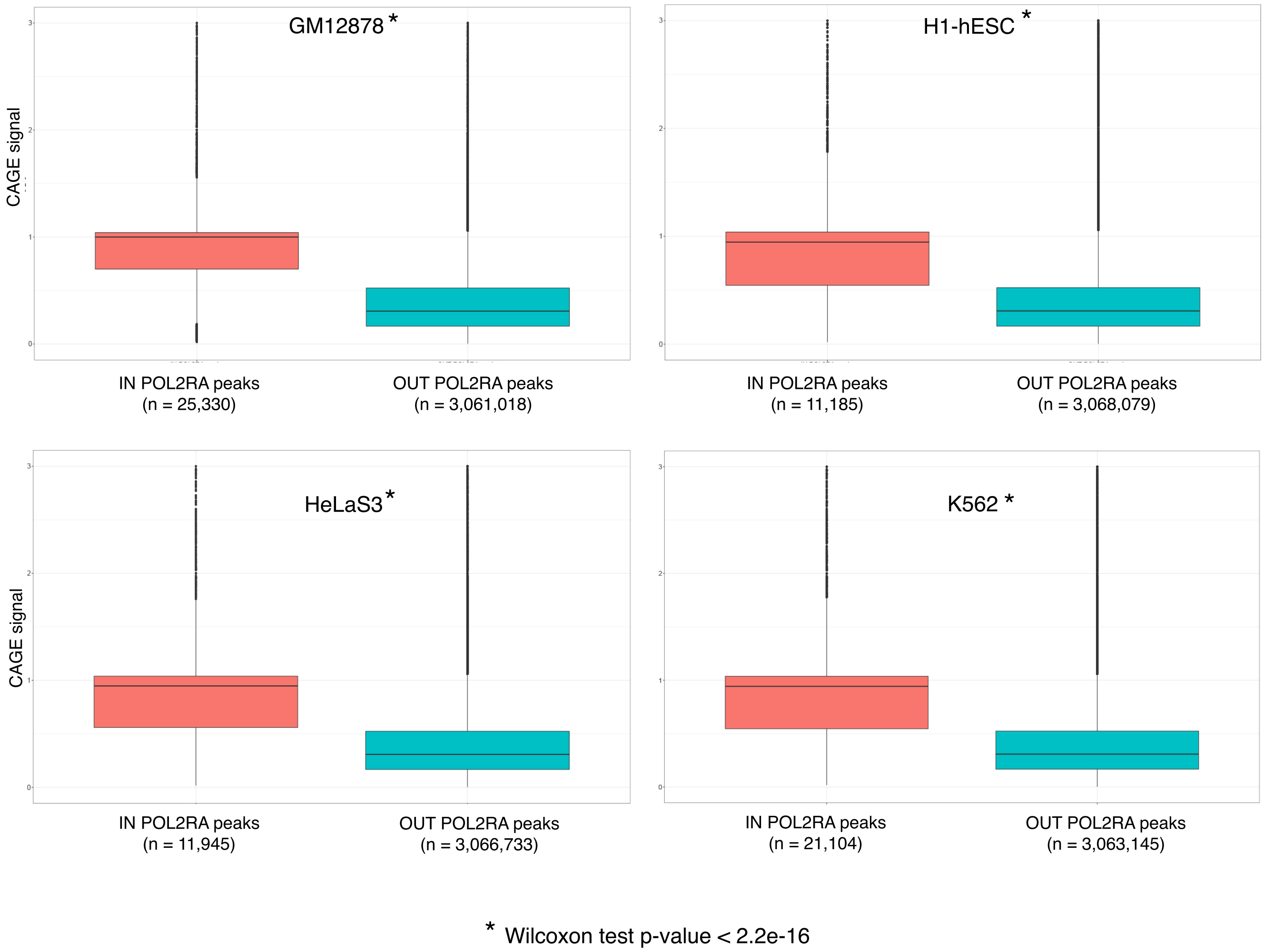

Supplementary Figure 5 – **CAGE signal at STRs located within RNAP-II peaks.** The coordinates of STRs were intersected with that of RNAP-II ChIP-seq narrow peaks from ENCODE. The CAGE signal associated with STRs located (red) or not (blue) in RNAPII binding sites were compared. Boxplots are defined as in Figure 1d. Two-sided Wilcoxon test was performed in all four cell types tested and the p-values were invariably < 2.2e-16.

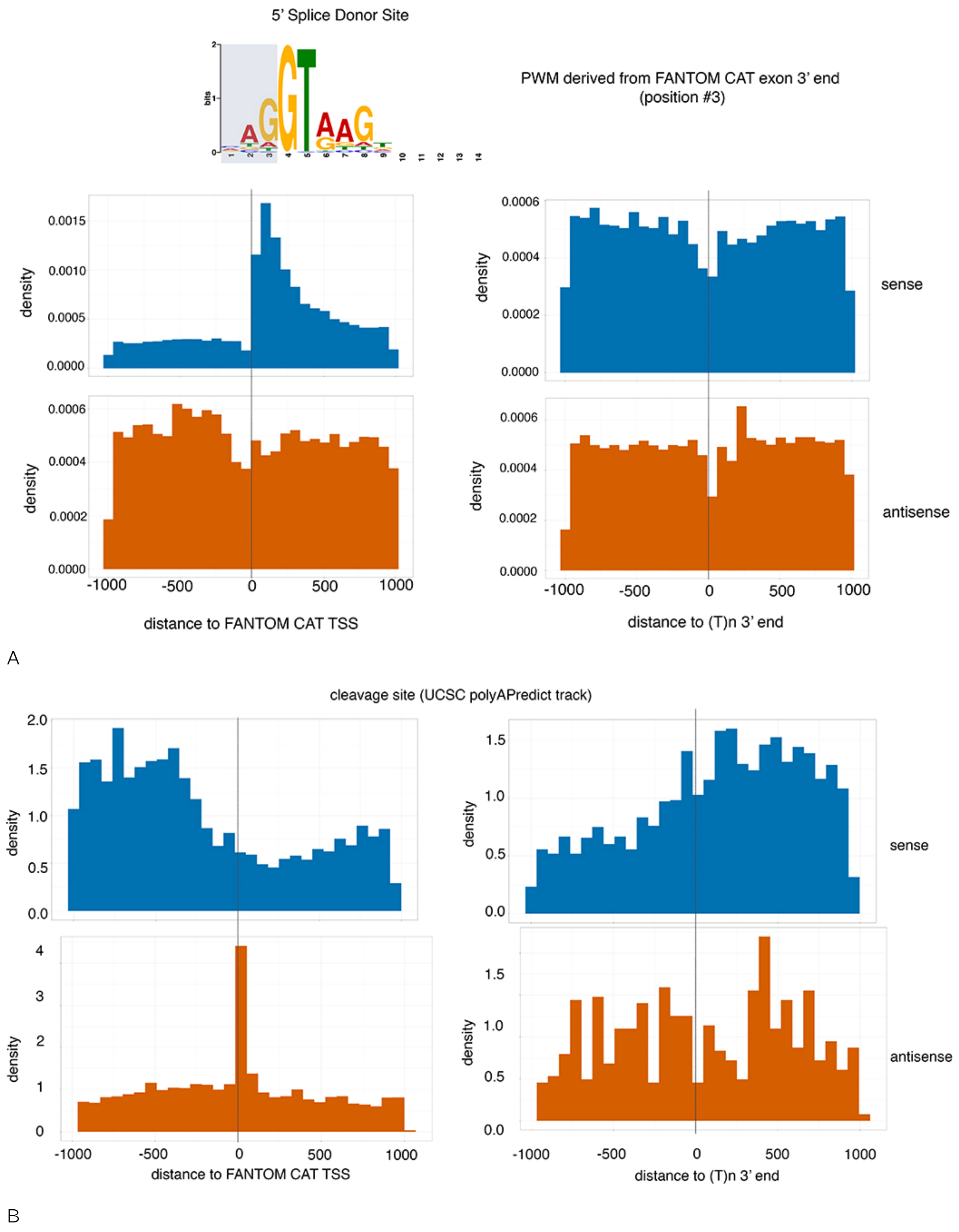

Supplementary Figure 6 – **Features associated to transcription directionality [2] A. U1 binding sites.** U1 PWM was built using MEME [3] and sequences encompassing -3/+10bp around FANTOM CAT 5' donor splice sites (exon 3' end). We then used this PWM to scan, with FIMO [4], 2kb regions centered around $(T)_n$ 3' ends (top 50,000 with the highest CAGE signal) and FANTOM CAT TSSs. **B. PolyA sites.** We used the UCSC track corresponding to the predictions made by Cheng *et al.* [5], as a bed file and used it in bedtools intersect [6] to look at polyA site distribution in regions encompassing 1 kb around $(T)_n$ 3' ends (top 50,000 with the highest CAGE signal) and FANTOM CAT TSSs. PolyA sites are enriched downstream FANTOM CAT TSSs, looking at the antisense orientation (or upstream in the sense orientation), as previously reported [2]

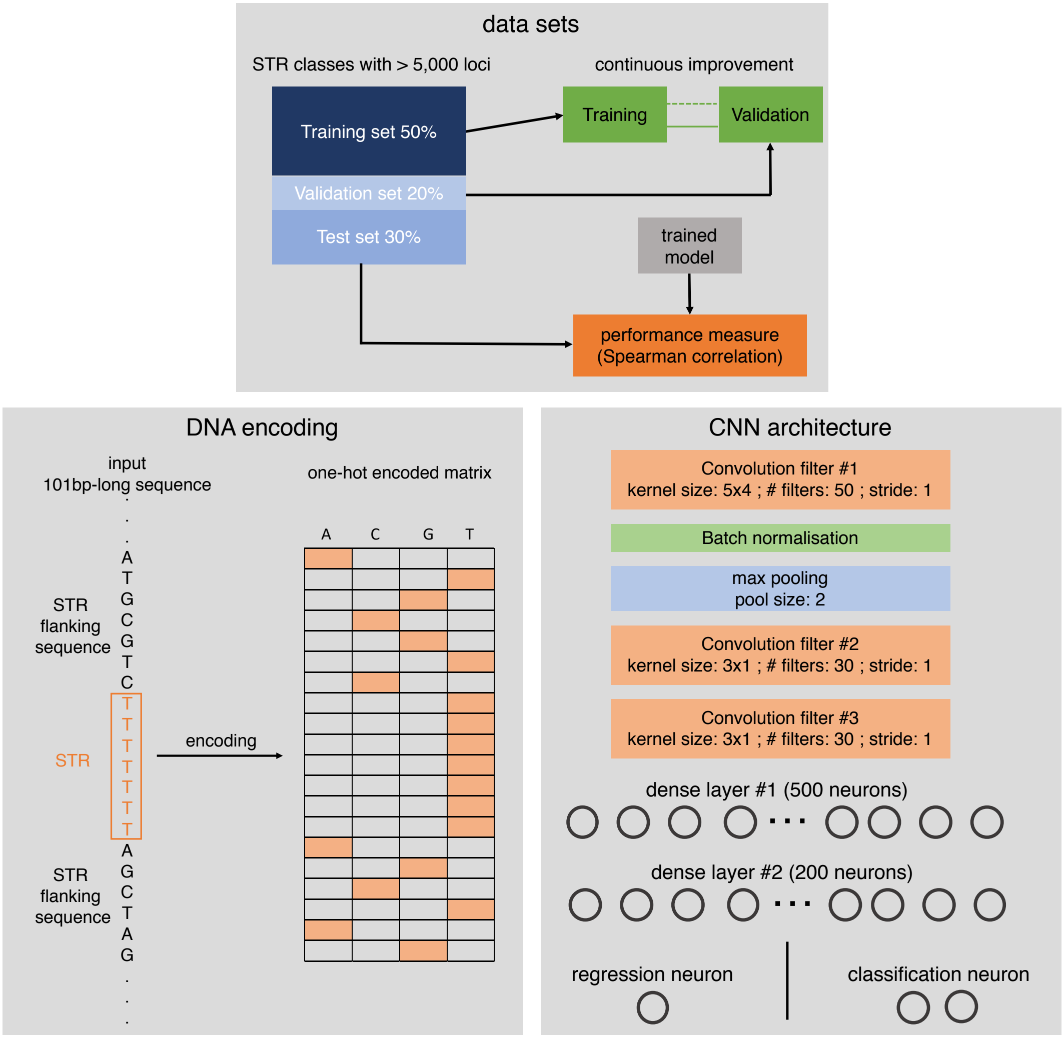

A

model (STR class)	# STRs in train set	# STRs in test set	# STRs with a CAGE peak in train set	# STRs with a CAGE peak in test set
T	522701	224015	47204	21720
A	549240	235389	943	447
AAAAC	25857	11082	43	15
AAAT	53343	22862	20	6
AC	97061	41598	1068	347
AG	36456	15624	241	79
AGG	4460	1912	264	94
AT	108145	46349	159	61
ATT	20110	8619	22	8
CT	36872	15803	564	215
CTTTT	10353	4438	300	163
GT	97245	41677	544	218
GTTT	38745	16606	79	34
GTTTTT	12810	5490	173	88

B

Supplementary Figure 7 – **A. Definition of testing/training sets, DNA encoding and model architectures.** The input sequence corresponds to ± 50bp around STR 3'end. Each layer is complemented with a RELU activation function, and dropout is implemented after the first dense layer. The model is either used for a classification (right) or a regression (left) task. Source code is available at `https://gite.lirmm.fr/ibc/deepSTR`. **B. number of sequences in train and test sets for the indicated model.**

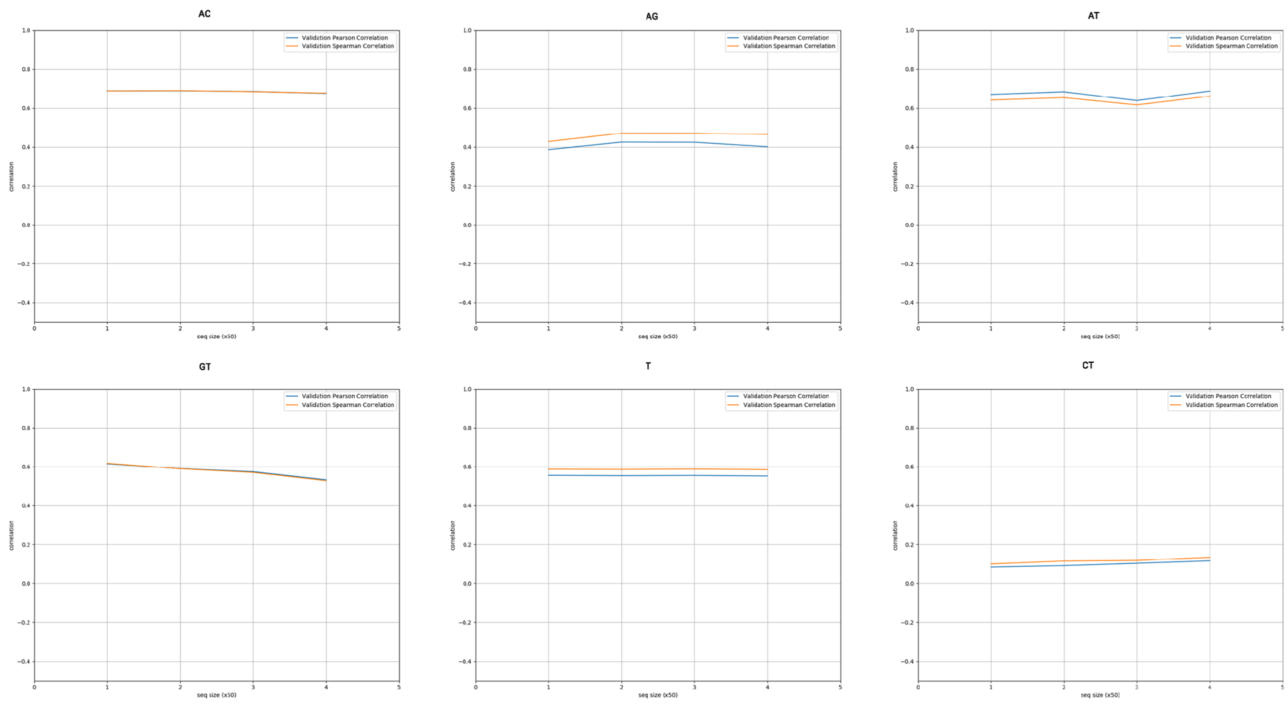

Supplementary Figure 8 – **Impact of the length of the sequences used as input of the CNN models.** Spearman (orange) and Pearson (blue) correlations (y-axis)were computed between the predicted and the observed CAGE signal. Different sequence size were tested as input (50bp, 100bp, 150bp and 200bp). The size is indicated as multiples of 50bp on the x-axis. Only 6 representative STR classes are shown.

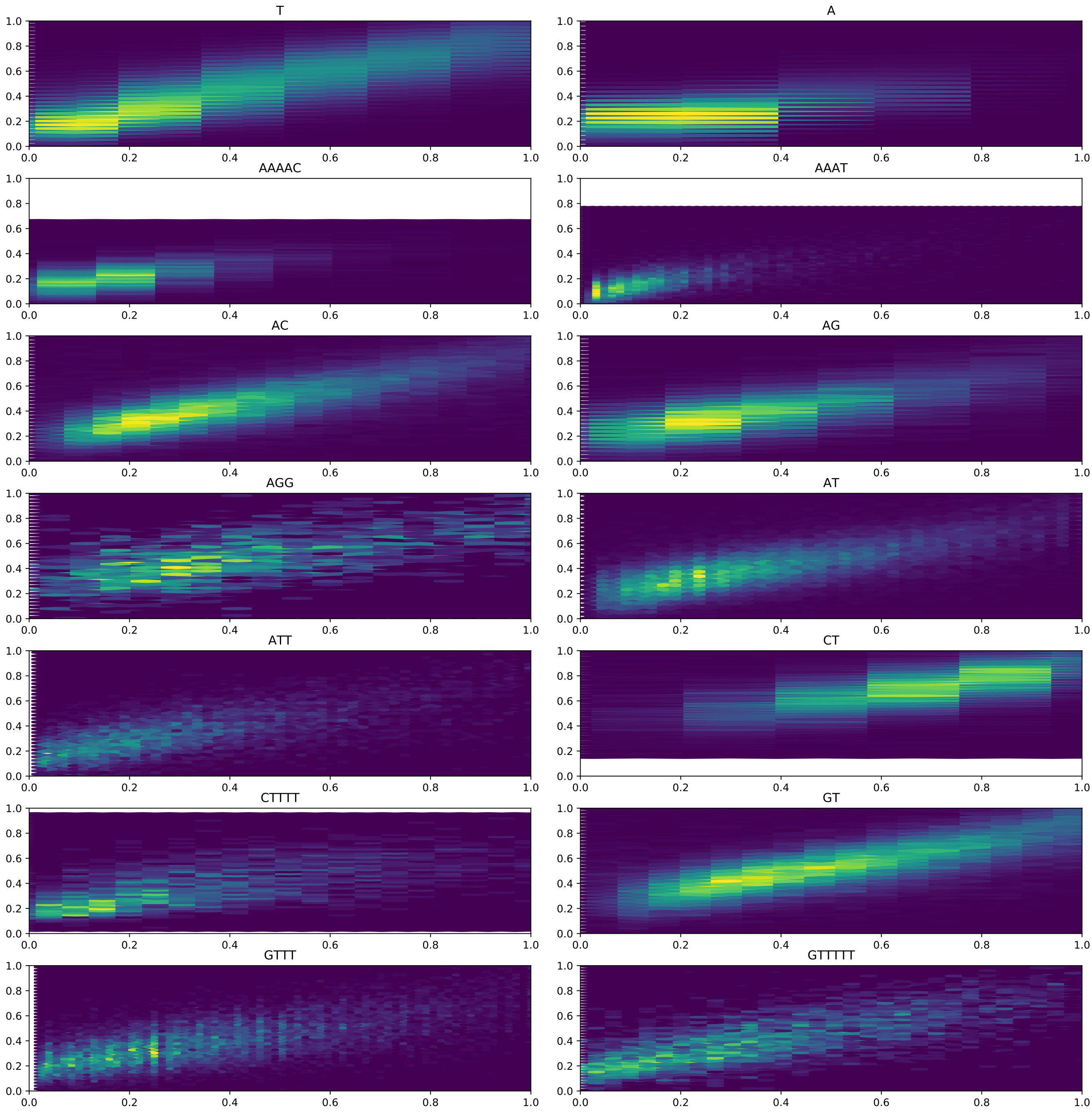

Supplementary Figure 9 – **Hexbin plots showing observed CAGE signal (x-axis) and signal predicted by class-specific models (y-axis) at STR classes shown in Figure 5A**. The STR class is indicated at the top of each plot. The color indicates the number of STRs considered from low (blue) to high (yellow).

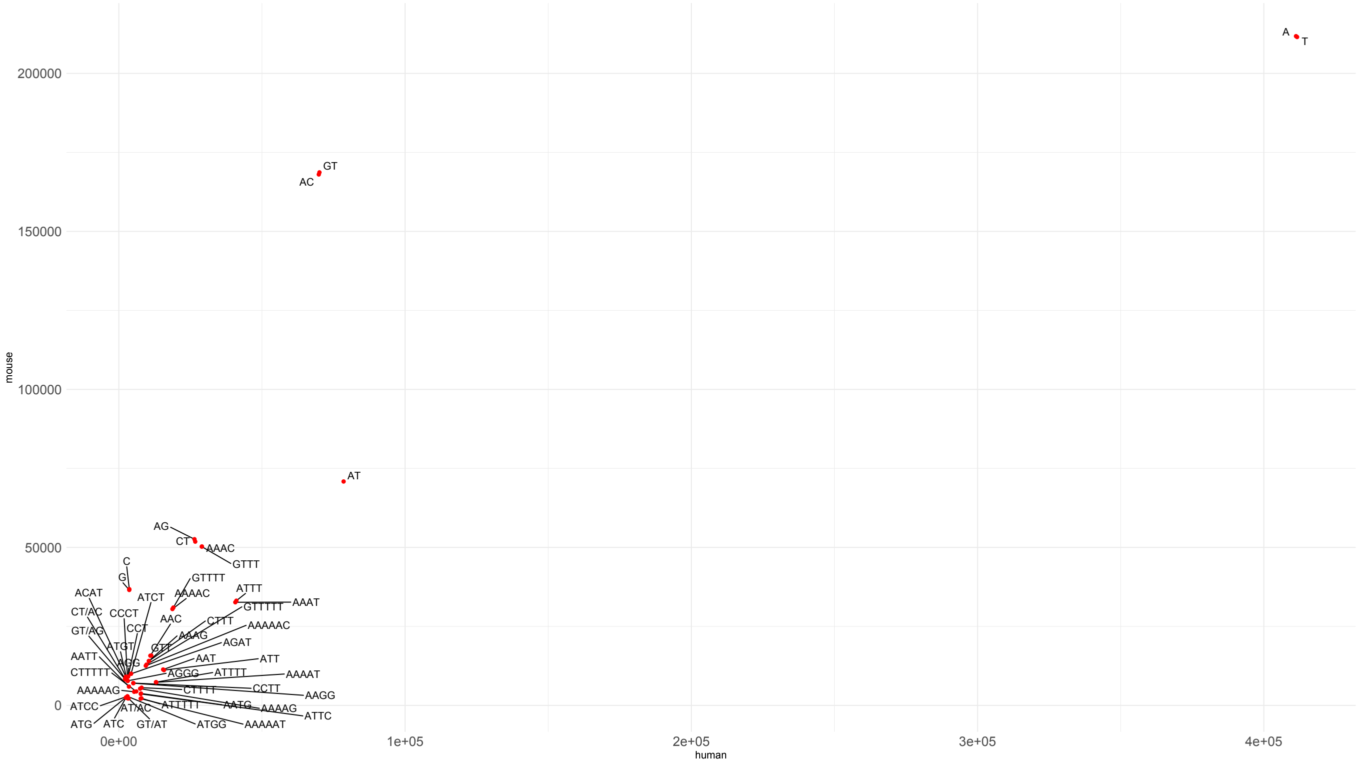

Supplementary Figure 10 – **Comparison of the number of loci within each STR class in human (x-axis) and mouse (y-axis) HipSTR catalogs**. Only STR classes with > 2,000 loci in human are shown for sake of clarity. The *ggrepel* R library was used.

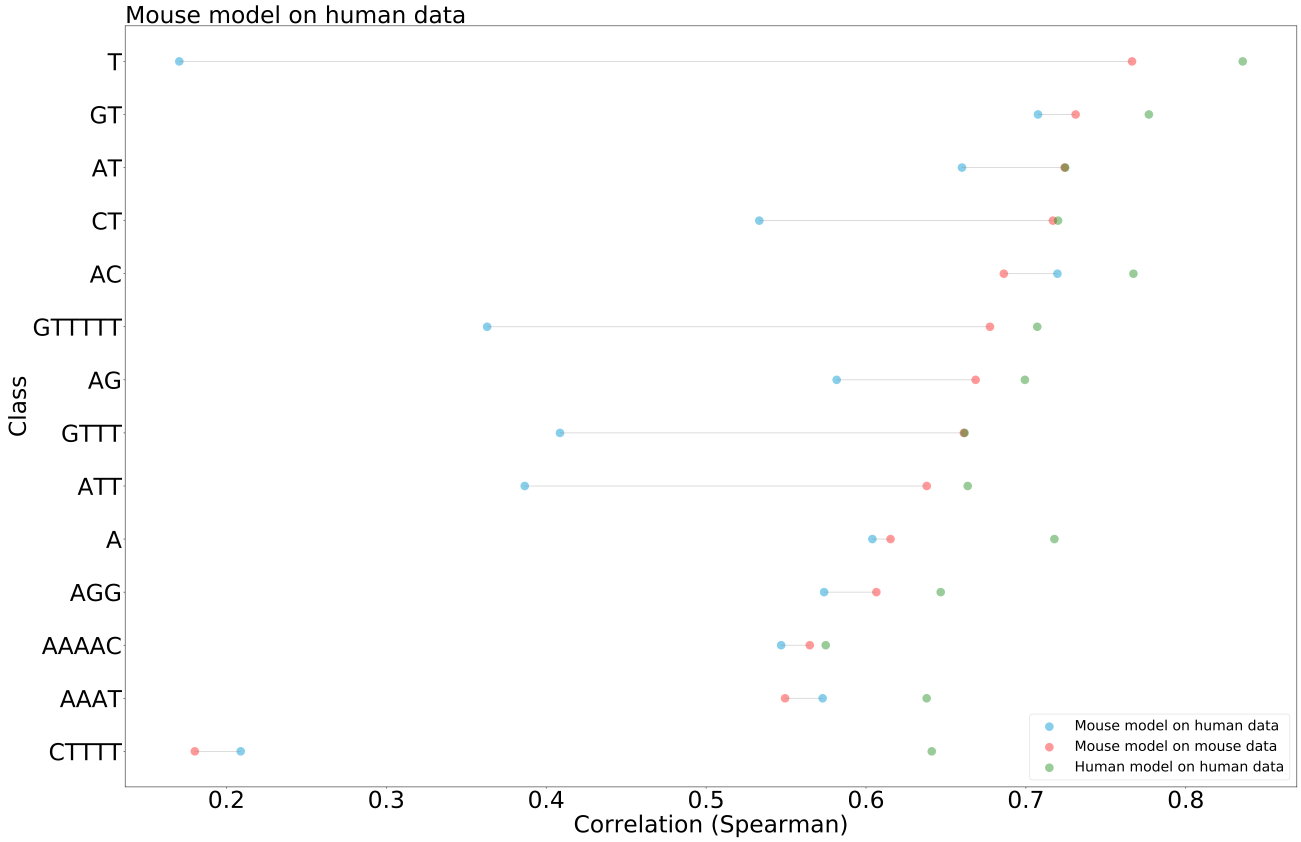

Supplementary Figure 11 – **Testing the accuracy of CNN models built in mouse and tested in human for different STR classes.** Performances of the models are assessed by computing the Spearman correlation between (i) CAGE signal observed in human and signal predicted by a model learned in mouse (blue dots), (ii) CAGE signal observed in mouse and signal predicted by a model learned in mouse (red dots) and (iii) CAGE signal observed in human and signal predicted by a model learned in human (green dots). The mouse models are overall less accurate than human models (Figure 6C). The $(CTTTT)_n$ mouse model performs poorly in mouse and human ($\rho < 0.2$). Likewise, this model hardly predicts transcription at human $(T)_n$ ($\rho < 0.2$). For other classes, the Spearman correlation between the signal predicted by the mouse model and the observed human signal was > 0.3, confirming that several features are conserved between human and mouse.

clnsig_1	clnsig_2	Mann-Whitney_pval
Pathogenic	Benign	1.84E-59
Pathogenic	other	9.85E-55
Conflicting_interpretations	other	1.30E-51
Benign/Likely_benign	other	3.41E-49
other	Likely_benign	2.85E-43
other	Uncertain_significance	6.13E-39
Conflicting_interpretations	Benign	5.52E-38
Pathogenic	Uncertain_significance	1.87E-32
Benign	Likely_benign	6.95E-32
Likely_pathogenic	other	2.88E-30
Benign/Likely_benign	Benign	1.06E-28
other	not_provided	8.43E-27
Benign	Uncertain_significance	4.74E-23
Benign	other	1.38E-22
Conflicting_interpretations	Uncertain_significance	2.81E-18
Pathogenic	Likely_benign	2.99E-16
Pathogenic/Likely_pathogenic	other	9.11E-16
Pathogenic	Likely_pathogenic	7.63E-13
Benign/Likely_benign	Uncertain_significance	2.51E-12
Likely_pathogenic	Conflicting_interpretations	1.16E-10
Conflicting_interpretations	Likely_benign	1.53E-10
Pathogenic	not_provided	1.59E-08
Benign/Likely_benign	Likely_pathogenic	5.41E-08
Conflicting_interpretations	not_provided	5.88E-08
Benign/Likely_benign	Likely_benign	6.41E-07
Likely_pathogenic	Benign	2.44E-06
Benign/Likely_benign	not_provided	2.47E-06
Uncertain_significance	Likely_benign	8.83E-06
Benign	not_provided	0.000318092
Conflicting_interpretations	Pathogenic/Likely_pathogenic	0.001552122
Pathogenic	Pathogenic/Likely_pathogenic	0.001746097
Benign/Likely_benign	Pathogenic/Likely_pathogenic	0.005280716
Likely_pathogenic	Likely_benign	0.01482522
Pathogenic/Likely_pathogenic	Benign	0.014967926
not_provided	Likely_benign	0.041768359
Pathogenic/Likely_pathogenic	Likely_benign	0.241828939

Supplementary Figure 12 – **Pairwise comparison of CAGE signal at STRs associated with ClinVar variants according to their clinical significance**. (related to Figure 7B) One-sided Mann-Whitney rank test p-values are indicated.

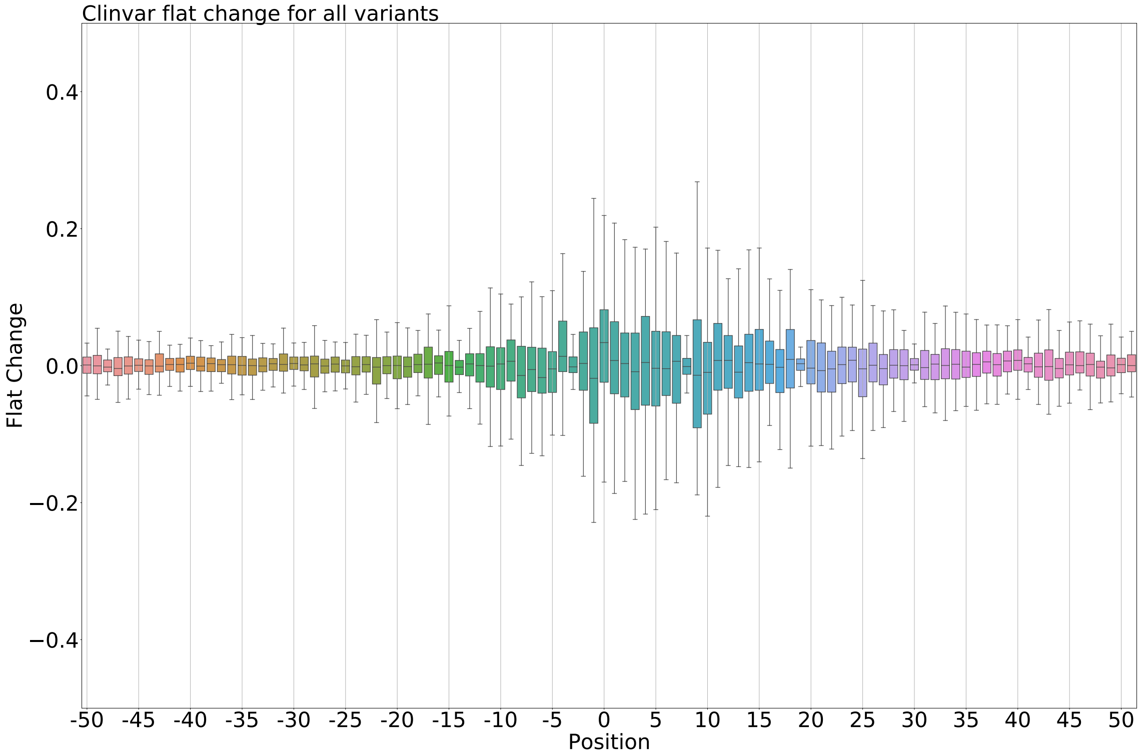

Supplementary Figure 13 – **Impact of ClinVar variants on CNN predictions.** Predictions are made on the hg19 reference sequence and on a mutated sequence, containing the genetic variants. Note that to keep sequences aligned, only single nucleotide variants are considered. Changes (y-axis) are measured as the difference between these two predictions (reference - mutated). Values are grouped by the position of the variants relative to the STR 3' end (position 0 on the x-axis). Note that variations at -3, 8 and 19 have no impact, revealing the potential existence of 'blind' positions, where models did not learn features.

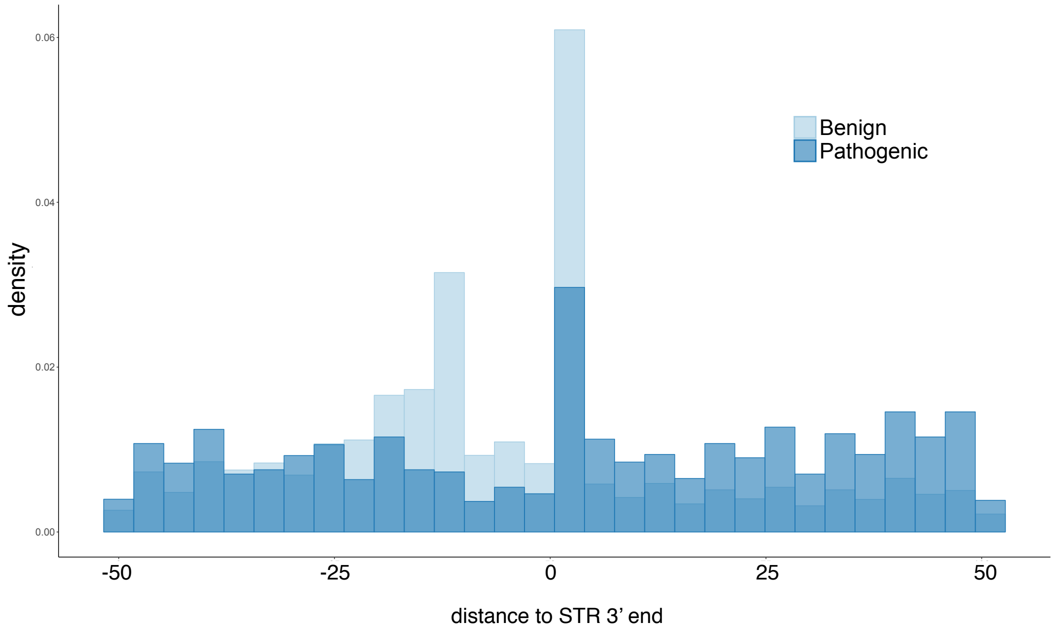

A

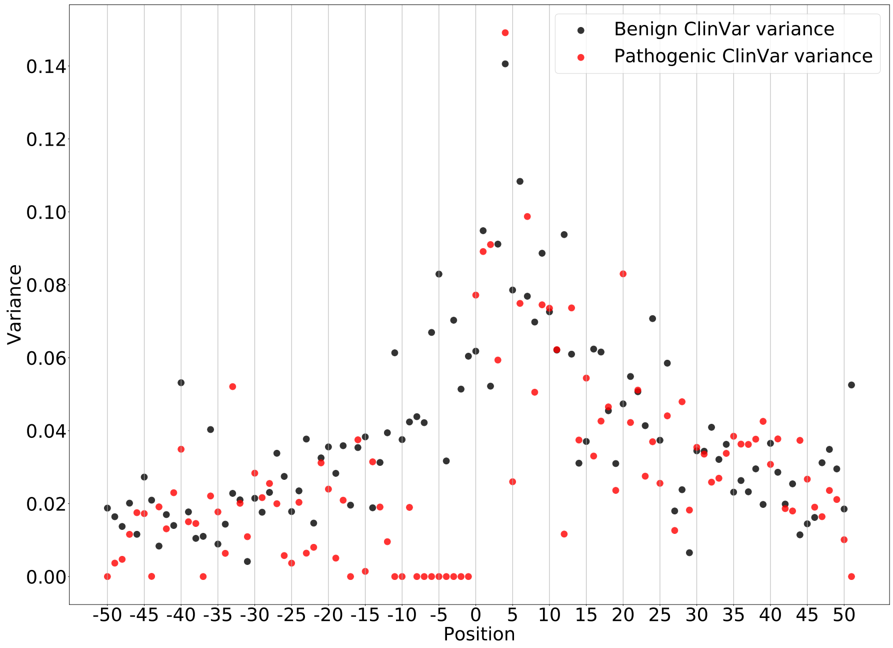

B

Supplementary Figure 14 – **A. Distribution of ClinVar benign and pathogenic variants around STR 3' end. B. Impact of ClinVar benign and pathogenic variants on CNN predictions.** Calculations are similar to that used in Figure 7C. Note that very few pathogenic variants are detected between -11 and 0 explaining why variance is close to 0 at these positions.

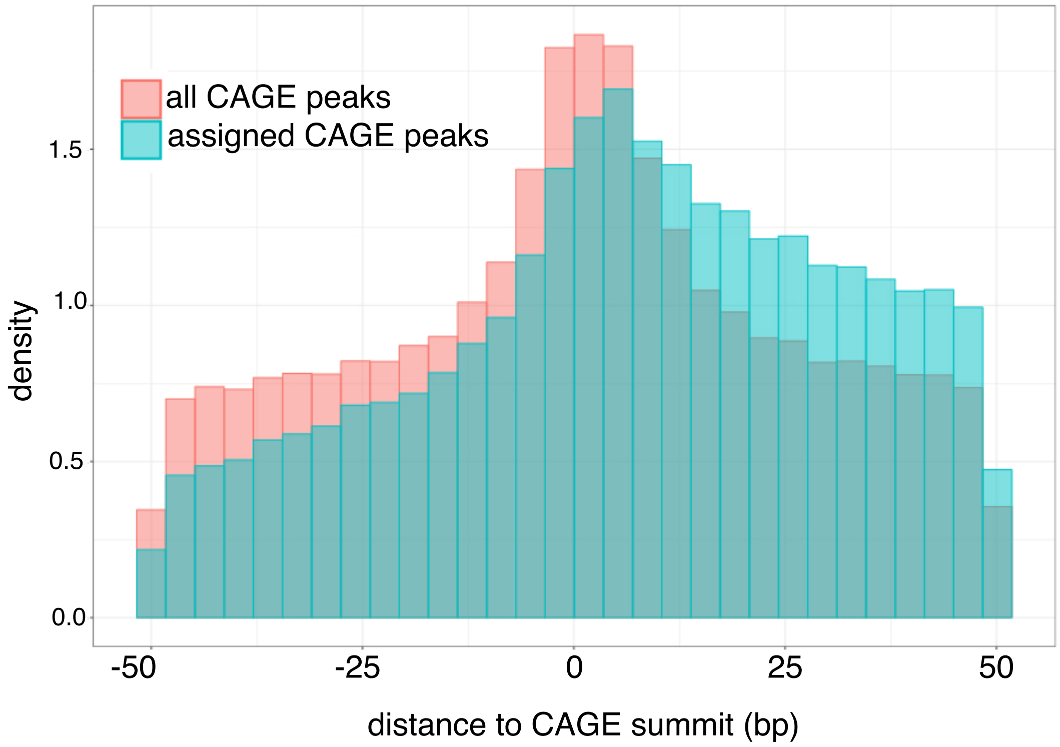

Supplementary Figure 15 – **Distribution of ClinVar variants around all CAGE peak summits (red, n = 1,048,124) and CAGE peak summits assigned to genes (blue, n = 130,286).**

STR_id	prediction_error	eQTL_slope	prediction	test
Human_STR_1013308;A;-	0.512843502	-0.274302	-0.023678958	True
Human_STR_1014538;A;+	0.15696609	0.94803	0.018923789	True
Human_STR_1025056;A;+	0.31890212	0.515692	0.006775588	True
Human_STR_104887;AG;+	0.685785919	-0.755728	-0.053272158	True
Human_STR_104887;AG;+	0.685785919	-0.853372	-0.053272158	True
Human_STR_1051947;A;+	0.134263456	-0.322115	0.058051974	False
Human_STR_1057874;T;-	0.355652475	-0.414139	-0.035879016	True
Human_STR_1098008;T;+	0.166469578	0.581679	-0.003184438	False
Human_STR_114278;T;-	0.594639405	1.18751	-0.000853896	False
Human_STR_1145023;A;-	0.542710841	0.33933	-0.08609882	False
Human_STR_1152088;T;+	0.153064203	-0.432745	0.047543645	False
Human_STR_1163037;T;-	0.271184408	-0.805364	-0.0267483	True
Human_STR_1179801;A;-	0.383187175	-0.304046	-0.046847194	True
Human_STR_1179982;A;-	0.060535314	0.202267	0.008506924	True
Human_STR_1186257;A;+	0.02233848	0.859361	0.001252085	True
Human_STR_1203266;A;+	0.036540616	0.720047	0.003545135	True
Human_STR_1211345;T;-	0.681278243	-1.82422	-0.012393713	True
Human_STR_1221313;GT;+	0.114279487	-1.34409	0.098219842	False
Human_STR_1224412;A;+	0.248036088	-0.981356	-0.047456533	True
Human_STR_1253679;AG;-	4.163502733	-0.337211	-0.031040907	True
Human_STR_1339136;A;+	0.324050128	-0.858856	-0.006126195	True
Human_STR_1352151;A;+	0.222833558	-0.531815	-0.013536438	True
Human_STR_1352981;A;-	0.550462081	-0.563385	0.008345604	False
Human_STR_1394791;AG;+	0.527901785	0.574247	-0.062838227	False
Human_STR_1395909;GT;-	0.122125666	0.436703	0.005783379	True
Human_STR_1397745;T;-	0.520498601	-0.973238	-0.040387392	True
Human_STR_1420016;A;-	0.04978914	0.506389	0.045680404	True
Human_STR_1428918;A;+	0.02320014	0.634781	0.003313512	True
Human_STR_1477782;T;-	0.419292437	-0.0985015	0.002373099	False
Human_STR_1498695;T;-	0.40177302	-0.464467	0.029380798	False
Human_STR_1520997;A;-	0.044008037	-0.413636	0.00636296	False
Human_STR_1577740;T;+	0.193279552	0.237593	0.029453993	True
Human_STR_177363;AC;+	0.22416	-0.879923	-0.009402037	True
Human_STR_244501;A;+	0.348763591	-0.486133	0.002373844	False
Human_STR_265539;A;+	0.001313722	0.406003	0.04482913	True
Human_STR_266013;T;-	0.543984513	-0.708419	0.002812862	False
Human_STR_269194;T;+	0.4035182	-1.09306	0.034630299	False
Human_STR_274217;A;-	0.230567726	0.539422	-0.000476301	False
Human_STR_278195;A;-	0.075378239	-0.945836	-0.002578676	True
Human_STR_306789;A;-	0.213919627	1.05788	-0.079474479	False
Human_STR_342681;A;+	0.028077471	-0.19582	-0.000938475	True
Human_STR_370230;A;-	0.370035506	-0.391885	0.026367664	False
Human_STR_429194;A;-	0.205172771	-0.259825	0.012718812	False
Human_STR_429319;AT;+	0.055873826	0.307993	0.012074232	True
Human_STR_449519;T;+	0.236857698	0.881822	-0.041200757	False
Human_STR_469778;T;+	0.29471579	-0.289375	0.118964911	False
Human_STR_469779;T;+	0.08052227	0.17614	0.152750969	True
Human_STR_481511;AC;-	0.182641478	-0.526342	0.022452414	False
Human_STR_501784;T;-	0.68943511	0.303861	-0.030791163	False
Human_STR_502546;A;-	0.210601421	-0.144698	0.001135856	False
Human_STR_507064;A;+	0.044711127	-0.661454	-0.028745979	True
Human_STR_534199;AC;-	0.094431053	0.142218	0.054202497	True
Human_STR_546583;A;-	0.326760536	-1.37129	-0.021778464	True
Human_STR_556777;T;+	0.084259953	-0.360379	0.032201022	False
Human_STR_560018;GT;-	0.59602199	0.323285	0.04092139	True
Human_STR_58127;A;+	0.101025903	-0.247657	-0.006968826	True
Human_STR_581987;A;-	0.103818262	-0.323734	-0.002291381	True
Human_STR_59182;A;-	0.71266678	-0.617571	0.001307279	False
Human_STR_595314;T;+	0.014739484	-0.593373	-0.108632192	True
Human_STR_595649;A;-	2.354146091	0.500722	-0.004173473	False
Human_STR_609609;T;-	0.546074089	-0.315424	0.007424593	False
Human_STR_610864;GT;+	0.083320588	-0.180904	-0.129508376	True
Human_STR_610864;GT;+	0.083320588	-0.266342	0.029965997	False
Human_STR_614935;A;-	0.245259867	-0.423531	-0.027294755	True
Human_STR_643252;GT;+	0.381810585	-0.33577	0.006754756	False
Human_STR_65688;T;-	0.501751912	-0.304512	0.009037495	False
Human_STR_657387;GT;-	0.432327108	0.385969	-0.036673963	False
Human_STR_672620;AC;+	0.131172236	0.328685	-0.013827205	False
Human_STR_683228;AC;+	0.148402994	0.560844	-0.03969416	False
Human_STR_690133;T;-	0.714274201	-0.411338	-0.01573348	True
Human_STR_695764;T;+	0.167998176	0.220017	0.018095374	True
Human_STR_697686;CT;-	0.250496686	0.921386	0.001309037	True
Human_STR_702647;T;-	0.705206527	-0.551792	-0.124134898	True
Human_STR_702835;A;-	0.776049972	-0.415228	0.004019678	False
Human_STR_729754;A;-	0.9502689	1.08792	0.004664108	True
Human_STR_73806;T;+	0.163897569	0.102133	0.003302217	True
Human_STR_745888;A;-	0.420911282	-0.241154	0.014324069	False
Human_STR_77791;A;-	0.398520128	-0.646491	-0.002977714	True
Human_STR_836210;T;+	0.136783695	-0.63492	-0.039980561	True
Human_STR_842539;T;+	0.129839683	-0.243308	-0.011493683	True
Human_STR_857035;A;+	0.081605225	0.51837	-0.061023772	False
Human_STR_85999;CT;-	0.480404344	-0.557144	-0.001500368	True
Human_STR_877237;A;-	0.346638614	-0.675917	0.009782106	False
Human_STR_896543;T;-	0.441590667	-0.218511	-0.044305921	True
Human_STR_917101;A;+	0.101821914	-0.356239	-0.035135686	True
Human_STR_932166;A;+	0.056582058	-0.408719	0.015662014	False

Supplementary Figure 16 – **Comparing CNN predictions and eQTLs.** (related to Supplementary Figure S17) prediction_error: absolute value of the difference between observation and prediction in the case of reference genome ; eQTL_slope: as computed by GTEx ; prediction: prediction(alternative allele) - prediction(reference) ; test: True if sign of eQTL slope = sign of prediction, False otherwise. Source code is available at `https://gite.lirmm.fr/ibc/deepSTR`.

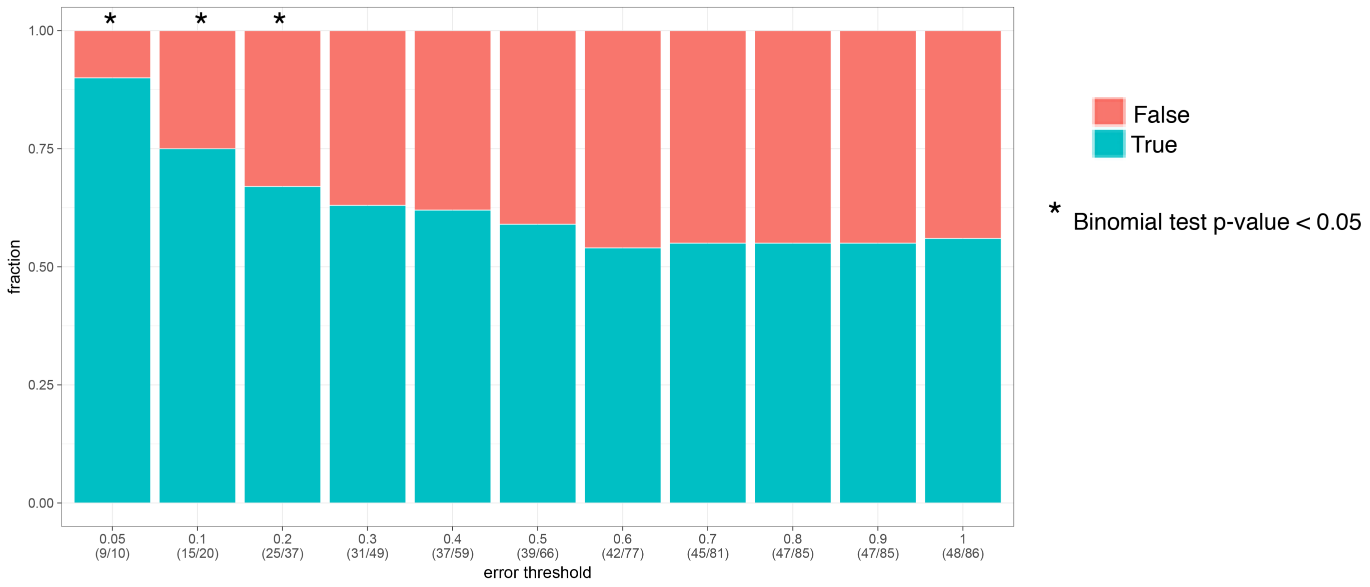

Supplementary Figure 17 – **Comparing CNN predictions and eQTLs.** (related to Supplementary Figure S16) Stacked plots showing the fraction of number of times CNN predictions and eQTL slopes are in agreement (True, blue) or not (False, red) (y-axis) for different prediction error thresholds (x-axis). The numbers in brackets indicate the number of eQTLs tested. Binomial tests were used to assess statistical relevance. Details of the calculations are provided in Supplementary Figure S16. See also text for details.

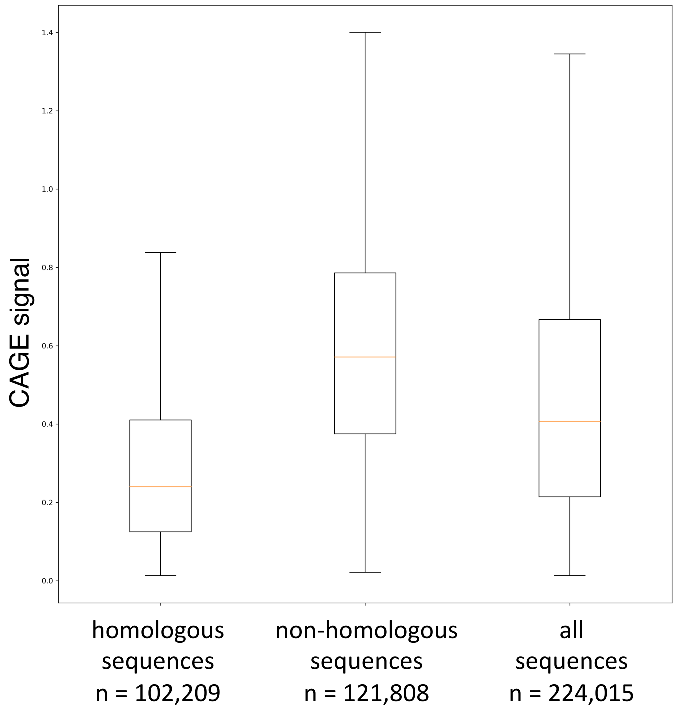

Supplementary Figure 18 – **CAGE signal in** $(T)_n$ **model test set.** (related to 'Convolutional Neural Network' in the Methods section). Homologous sequences : sequences from the test set with > 60% query cover and > 80% identity with sequences from the train set, according to BLASTn (n = 102,209). All sequences: whole test set (n = 224,015) ; Non-homologous sequences: whole test set - homologous sequences (n = 121,808). Boxplots are defined as in Figure 1d except that points beyond the end of the whiskers are not plotted for clarity. One-way Anova test was used to assess overall statistical differences (p-value < 2.2e-16).

5' linker GN5 up	5’- GTGGTAUCAACGCAGAGUACGNNNNN -P-3'
5' linker N6 up	5’- GTGGTAUCAACGCAGAGUACNNNNNN -P-3'
5' linker down	5'-P- GTACTCTGCGTTGATACCAC-P-3'
3' linker up	5'-AAAAABBBBBBBBGCAUCGCUGTCTCUTAUACACAUCUCCGAGCCCACGAGAC -P-3'
3' linker down	5'- GTCTCGTGGGCTCGGAGATGTGTATAAGAGACAGCGATGC -3'
2nd primer	5’- TCGTCGGCAGCGTCAGATGTGTATAAGAGACAGNNNNNNNNGTGGTATCAACGCAGAGTAC -3’

Supplementary Table 1 – **Primers used for MinION sequencing.**

References

[1] Hon, C. C. *et al.* An atlas of human long non-coding RNAs with accurate 5’ ends. *Nature* **543**, 199–204 (2017).

[2] Almada, A. E., Wu, X., Kriz, A. J., Burge, C. B. & Sharp, P. A. Promoter directionality is controlled by u1 snrnp and polyadenylation signals. *Nature* **499**, 360–363 (2013).

[3] Bailey, T. L., Elkan, C. *et al.* Fitting a mixture model by expectation maximization to discover motifs in bipolymers (1994).

[4] Grant, C. E., Bailey, T. L. & Noble, W. S. Fimo: scanning for occurrences of a given motif. *Bioinformatics* **27**, 1017–1018 (2011).

[5] Cheng, Y., Miura, R. M. & Tian, B. Prediction of mrna polyadenylation sites by support vector machine. *Bioinformatics* **22**, 2320–2325 (2006).

[6] Quinlan, A. R. & Hall, I. M. BEDTools: a flexible suite of utilities for comparing genomic features. *Bioinformatics (Oxford, England)* **26**, 841–842 (2010).
