# Supplementary material for: Discovery of widespread transcription initiation at microsatellites predictable by sequence-based deep neural network
Source: Nat Commun. 2021 Jun 2;12:3297. doi: 10.1038/s41467-021-23143-7 (PMC8172540; doi:10.1038/s41467-021-23143-7)
Supplement: Supplementary file 3 — Reporting Summary [file 41467_2021_23143_MOESM3_ESM.pdf]

nature research

Corresponding author(s): LECELLIER - NCOMMS-20-28357-T

Last updated by author(s): 2021/02/24

Reporting Summary

Nature Research wishes to improve the reproducibility of the work that we publish. This form provides structure for consistency and transparency in reporting. For further information on Nature Research policies, see our Editorial Policies and the Editorial Policy Checklist.

Statistics

For all statistical analyses, confirm that the following items are present in the figure legend, table legend, main text, or Methods section.

n/a	Confirmed	
☐	☒	The exact sample size (*n*) for each experimental group/condition, given as a discrete number and unit of measurement
☐	☒	A statement on whether measurements were taken from distinct samples or whether the same sample was measured repeatedly
☐	☒	The statistical test(s) used AND whether they are one- or two-sided<br>*Only common tests should be described solely by name; describe more complex techniques in the Methods section.*
☐	☒	A description of all covariates tested
☐	☒	A description of any assumptions or corrections, such as tests of normality and adjustment for multiple comparisons
☐	☒	A full description of the statistical parameters including central tendency (e.g. means) or other basic estimates (e.g. regression coefficient) AND variation (e.g. standard deviation) or associated estimates of uncertainty (e.g. confidence intervals)
☐	☒	For null hypothesis testing, the test statistic (e.g. *F*, *t*, *r*) with confidence intervals, effect sizes, degrees of freedom and *P* value noted<br>*Give P values as exact values whenever suitable.*
☒	☐	For Bayesian analysis, information on the choice of priors and Markov chain Monte Carlo settings
☐	☒	For hierarchical and complex designs, identification of the appropriate level for tests and full reporting of outcomes
☐	☒	Estimates of effect sizes (e.g. Cohen's *d*, Pearson's *r*), indicating how they were calculated

Our web collection on statistics for biologists contains articles on many of the points above.

Software and code

Policy information about availability of computer code

Data collection	Data sources (url) are indicated in the Methods section. CTR-seq data were deposited on DNA Data Bank of Japan Sequencing Read Archive (accession number: DRA010491). Processed data are available at https://gite.lirmm.fr/ibc/deepSTR.
Data analysis	Source code of the models, a readme.txt file and other instructions for installing and running the analyses are available at https://gite.lirmm.fr/ibc/deepSTR.

For manuscripts utilizing custom algorithms or software that are central to the research but not yet described in published literature, software must be made available to editors and reviewers. We strongly encourage code deposition in a community repository (e.g. GitHub). See the Nature Research guidelines for submitting code & software for further information.

Data

Policy information about availability of data

All manuscripts must include a data availability statement. This statement should provide the following information, where applicable:

- Accession codes, unique identifiers, or web links for publicly available datasets
- A list of figures that have associated raw data
- A description of any restrictions on data availability

The urls of the datasets generated during and/or analysed during the current study are indicated in the Methods section and available at https://gite.lirmm.fr/ibc/deepSTR.

Field-specific reporting

Please select the one below that is the best fit for your research. If you are not sure, read the appropriate sections before making your selection.

[x] Life sciences [ ] Behavioural & social sciences [ ] Ecological, evolutionary & environmental sciences

For a reference copy of the document with all sections, see nature.com/documents/nr-reporting-summary-flat.pdf

Life sciences study design

All studies must disclose on these points even when the disclosure is negative.

Sample size	No sample-size calculation was performed. All STRs listed in the HipSTR catalogs (https://github.com/HipSTR-Tool/HipSTR-references/raw/master/human/hg19.hipstr_reference.bed.gz and https://github.com/HipSTR-Tool/ HipSTR-references/blob/master/mouse/mm10.hipstr_reference.bed.gz) were used along with all the data resources used in the current study (see Methods section).
Data exclusions	No data was excluded from the study.
Replication	Two libraries of CTR-seq were generated (with and without polyA tailing), and the results were consistent as shown Figure 3. For computational analyses, because all data were used, no replication was required. For deep learning, we separated training, testing and validation datasets prior to model training, and these sets were stored on disk (Figure S7). This allowed us to carry analyses on held-out data that has never been seen by the models. We also made sure that our models do not overfit due for instance to homologous sequences present in both train and test sets (see Methods section). All attempts at replicating our models (at least 10) were consistent and successful.
Randomization	As indicated in the Methods section, all STR classes of the HipSTR catalogs with > 5,000 elements were analyzed. Assignements to training, testing and validation datasets were done randomly using Pytorch SubsetRandomSampler.
Blinding	For deep learning analyses, all analyses were performed on held-out test sets to avoid optimistic bias in accuracy estimation. For other analyses, blinding is not relevant.

Reporting for specific materials, systems and methods

We require information from authors about some types of materials, experimental systems and methods used in many studies. Here, indicate whether each material, system or method listed is relevant to your study. If you are not sure if a list item applies to your research, read the appropriate section before selecting a response.

Materials & experimental systems

n/a	Involved in the study	
[x]	[ ]	Antibodies
[ ]	[x]	Eukaryotic cell lines
[x]	[ ]	Palaeontology and archaeology
[x]	[ ]	Animals and other organisms
[x]	[ ]	Human research participants
[x]	[ ]	Clinical data
[x]	[ ]	Dual use research of concern

Methods

n/a	Involved in the study	
[x]	[ ]	ChIP-seq
[x]	[ ]	Flow cytometry
[x]	[ ]	MRI-based neuroimaging

Eukaryotic cell lines

Policy information about cell lines

Cell line source(s)	ATCC A549
Authentication	A549 cell line was purchased from ATCC along with a certified authentification.
Mycoplasma contamination	Cell lines were tested negative for mycoplasma contamination.
Commonly misidentified lines (See ICLAC register)	No commonly misidentified cell line was used.
