# Supplementary material for: Discovery of widespread transcription initiation at microsatellites predictable by sequence-based deep neural network
Source: Nat Commun. 2021 Jun 2;12:3297. doi: 10.1038/s41467-021-23143-7 (PMC8172540; doi:10.1038/s41467-021-23143-7)
Supplement: Supplementary file 4 — Description of Additional Supplementary Files [file 41467_2021_23143_MOESM4_ESM.pdf]

Description of Additional Supplementary Files

File Name: Supplementary Data 1
Description: coordinates of FANTOM CAT transcripts and enhancers whose transcription initiation sites are located at STRs.

File Name: Supplementary Data 2
Description: Enrichment of disease-related variants in STRs

File Name: Supplementary Data 3
Description: Enrichment of STR classes in FANTOM CAT biotypes
